# Supplementary material for: The spatiotemporal association of non-prescription retail sales with cases during the 2009 influenza pandemic in Great Britain
Source: BMJ Open. 2014 Apr 29;4(4):e004869. doi: 10.1136/bmjopen-2014-004869 (PMC4010812; doi:10.1136/bmjopen-2014-004869)
Supplement: Author's manuscript [file bmjopen-2014-004869.draft_revisions.pdf]

The spatio-temporal association of non-prescription retail sales with cases during the 2009 influenza pandemic in Great Britain.

Journal:	*BMJ Open*
Manuscript ID:	bmjopen-2014-004869
Article Type:	Research
Date Submitted by the Author:	16-Jan-2014
Complete List of Authors:	Todd, Stacy; Liverpool School of Tropical Medicine, Clinical Sciences; Oxford University Clinical Research Unit,<br>Diggle, Peter; University of Liverpool, Institute of Infection and Global Health; University of Lancaster, Lancaster Medical School<br>White, Peter; Imperial College, School of Public Health; Public Health England, Modelling and Economics Unit<br>Fearne, Andrew; University of Kent, Kent Business School<br>Read, Jonathan; University of Liverpool, Institute of Infection and Global Health
**Primary Subject Heading**:	Public health
Secondary Subject Heading:	Epidemiology, Infectious diseases
Keywords:	Influenza, Syndromic Surveillance, Outbreak

SCHOLARONE™
Manuscripts

For peer review only

The spatio-temporal association of non-prescription retail sales with cases during the 2009 influenza pandemic in Great Britain.

Stacy Todd[1,2], Peter J Diggle[3,4], Peter J White[5,6], Andrew Fearne[7], Jonathan M Read[3].

1. Dept of Clinical Sciences, Liverpool School of Tropical Medicine, UK
2. Oxford University Clinical Research Unit, Wellcome Trust Major Overseas Programme, Ho Chi Minh City, Vietnam
3. Institute of Infection and Global Health, University of Liverpool, UK
4. Lancaster Medical School, University of Lancaster, UK
5. School of Public Health, Imperial College, UK
6. Modelling and Economics Unit , Public Health England, UK
7. Kent Business School, University of Kent, UK

Corresponding Author:

Dr Jonathan M Read
Department of Epidemiology & Population Health,
Institute of Infection and Global Health,
Faculty of Health and Life Sciences,
University of Liverpool, Leahurst Campus,
Neston, South Wirral
United Kingdom CH64 7TE
E: jonread@liv.ac.uk
T: +44 151 794 6092
F: +44 151 794 6028

Key Words: Influenza; Syndromic Surveillance (MESH); Outbreak
Word Count: 2645

For peer review only

ABSTRACT

Background: As many individuals will self medicate for mild influenza-like illness, surveillance of non-prescription purchases may be an important adjunct to healthcare-based surveillance in early assessment of the severity of a novel influenza strain or other pathogen. Its usefulness as a marker of seasonal influenza has been investigated for over 30 years with varying degrees of success.

Objective: The aim of this paper was to compare spatio-temporal patterns of retail sales, influenza cases during the 2009 influenza pandemic.

Methods: Weekly, seasonally-adjusted sales by a major British supermarket of over-the-counter symptom remedies and non-pharmaceutical products; recommended as part of the advice offered by public health agencies; were compared with weekly influenza case estimates. Comparisons were made at national and regional spatial resolutions. We also compared sales to national measures of contemporaneous media output and public interest (internet search volume) related to the pandemic.

Results: At a national scale there was no significant correlation between retail sales of symptom remedies and cases for the whole pandemic period in 2009. At the regional scale, a minority of regions showed statistically significant positive correlations between cases and sales of adult 'cold and flu' remedies and cough remedies, but a greater number of regions showed a significant positive correlation between cases and symptomatic remedies for children. Significant positive correlations between cases and sales of thermometers and anti-viral hand gels/wash were seen at both spatial scales. We found no significant association between retail sales and media reporting or internet search volume.

Conclusion: This study provides evidence that the British public responded appropriately to health messaging about hygiene. Retail sales at a national level are not useful for the detection of cases. However, at finer spatial scales, in particular age-groups, retail sales may help augment existing surveillance and merit further study.

For peer review only

ARTICLE SUMMARY

Strengths and limitations of the study

- This study is the first to examine associations between non-prescription retail sales and influenza cases at different spatial resolutions in a British setting and in particular it's potential as part of syndromic surveillance systems.
- The adjustment for seasonality in retail sales was fitted for each spatial resolution to attempt to capture regional differences which may exist.
- The inclusion of non-pharmaceutical products allowed for the first objective assessment of the response to government public health messaging.
- The main weakness of this study is that regional data was available only for England and for a portion of the 2009/2010 pandemic period.
- Increasing the years of sales data prior to the pandemic period would have provided a more robust estimate of sales trend in a typical year.

For peer review only

Introduction

Public health surveillance has traditionally relied on healthcare providers reporting selected notifiable conditions, usually with biological confirmation [1]. Although a key part of national and international health regulations, this system has well-recognised problems including delays in reporting and difficulty in identifying unusual activity [2]. Expansion of non-traditional surveillance methods has occurred over the last 2 decades, initially because of concerns regarding bioterrorism, and has now been adopted into routine public health systems in many countries. These methods (often referred to as Syndromic Surveillance Systems) offer a real-time or near-real-time collection of data from a variety of sources, ideally in an automated manner which allows early identification of the spread and impact of potential public health threats [3]. The 2009 influenza pandemic provided the motivation to adopt and appraise many of these methods [4 5]. In the UK many of the lessons learned during this time were subsequently adopted during the 2012 Olympics and Paralympics to identify any early infectious disease threat [6].

The surveillance of infectious diseases can be strongly affected by the care seeking behaviour of individuals [7]. As many individuals will self-medicate for mild illness, surveillance of non-prescription sales has been suggested as an adjunct to healthcare based surveillance to estimate the magnitude and dynamics of care seeking behaviour [8]. Its usefulness for surveillance of seasonal influenza [9-13] and other illnesses [14-17] has been examined for over 30 years with varying degrees of success. A major potential benefit of this type of surveillance system would be to provide more reliable estimates of incidence when the propensity to seek care is low or changeable, and to identify early-stage epidemics through unusual purchasing activity. At present, retail sales are not used for syndromic surveillance in Great Britain.

Here, we describe the temporal and spatial patterns of sales of over-the-counter flu and cold remedies and non-pharmaceutical products; recommended as part of the advice offered by public health agencies; sold by a major British supermarket, and compare these patterns to national, regional and sub-regional estimated cases of pandemic influenza during 2009 in Great Britain. We also compare the pattern of sales to national measures of media output and public interest (internet search volume) related to the pandemic during the same time period.

Methods

Data sources (Table 1)

The weekly estimates of influenza cases were obtained via the Health Protection Agency (HPA; now part of Public Health England) as part of their influenza surveillance systems [18]. UK-wide data was calculated via the FluSurvey project (www.flusurvey.org) which adjusted healthcare-based surveillance systems to account for changes in care-seeking behaviour during the pandemic, as determined though an online survey of a community cohort [19]. Regional case data was available through the HPA/Q-Surveillance network which monitors diagnoses recorded by general practitioners onto routine electronic systems and extracted on a daily and weekly basis [20]. Over 3,400 practices contribute to the system, which covers approximately 38% of the UK population; most of the practices are in England with fewer in Wales and Northern Island. At the time of the 2009 pandemic no Scottish practices contributed to the system. The density of coverage allows reporting at country and regional levels. Regionally this corresponds to 10 English Strategic Health Authorities (SHAs) and 156 Primary Care Trusts (PCTs), which is the lowest unit of healthcare

For peer review only

provision in England with an average population size of 350,000. The HPA/Q-Surveillance data was provided as daily counts of reported cases in each PCT and including estimated population in each PCT for that day. This was aggregated to a weekly scale and converted to incidence as a rate of cases per 100,000 population. HPA/QSurveillance data was aggregated to 3 spatial resolutions; sub-regional, regional and country level (corresponding to PCT, SHA and England, Wales and NI respectively).

Two measures of media interest and one of public interest over time were compiled. Daily national newspaper article counts were compiled from the Lexis Nexis newspaper archive [21], counting articles with headlines containing "swine flu" or "h1n1". The same search phrases were used to identify relevant articles on the Meltwater online database: this database includes newspaper, online, television and radio news articles and reporting [22]. Internet search trends were used as a proxy for public interest in the pandemic. This was derived from Google Insight search facility [23], and the daily relative volume of searches made where the search terms contained the terms "swine flu" or "h1n1" were collated.

Weekly unit sales of non-prescription retail products for a major national UK retailer were obtained for the period 28 January 2008 to 25 April 2010. These sales records were derived from a 10% sample of transactions where a loyalty card was presented at the point of purchase and were available at store level. Data on individual product sales were extracted from a master database and aggregated into six categories: Adult Cold and Flu Remedies; Children's Cold and Flu Remedies; Cough Remedies; Thermometers; Anti-Viral Products (including hand gel and wipes); Tissues. Sales as a proportion of customer base were used instead of absolute sales to control for confounders such as changes in store hours in the period of the study or variation in market share between stores. Short shelf-life products were assumed to be indicative of total customer base. Sales were therefore adjusted in the first instance by dividing weekly total sales (for each category of product and spatial scale) by the average weekly sales of milk and bananas at the appropriate spatial scale (annual sales for 2008 and 2009 available).

The extreme seasonality associated with influenza (and subsequently symptomatic remedies) in temperate zones could introduce biases in the analysis. To adjust for this, an underlying seasonal trend in proportional sales was fitted to log-transformed retail sales data from the beginning of February 2008 to the end of January 2009. This was a pre-pandemic year, which we assumed to be typical of the seasonal trend in influenza incidence. A flexible way to represent a seasonal trend is through a sum of sine-cosine waves with frequencies corresponding to 1, 2, 3, etc cycles per year. For example, the model with 2 sine-cosine pairs is

$$ln(y_t + 1) = \alpha_0 + \alpha_1 \sin\left(\frac{2\pi t}{52}\right) + \beta_1 \cos\left(\frac{2\pi t}{52}\right) + \alpha_2 \sin\left(\frac{4\pi t}{52}\right) + \beta_2 \cos\left(\frac{4\pi t}{52}\right) + \varepsilon_t$$

where y_t is the retail sales data for each week of the year, t, during 2008, α and β terms are the regression coefficients for each sine and cosine function, and ε is an error term.

The model-fitting process was repeated for each product category at each spatial resolution. This resulted in between 1 and 4 sine-cosine pairs across the different product groups. In each case, the fitted seasonal model was used to derive weekly residuals for each week of the 2009 and 2010 data;

For peer review only

these residuals, which are normalized with respect to normal non-pandemic seasonal sales, are used in the comparative analysis (Supplementary Appendix Table A1, Figure A1 and A2).

Pearson's correlation was performed between each product category, national UK cases and media reporting. Analysis was performed for the whole pandemic period as well as the early pandemic period (06 April- 01 June 2009, media reporting only), summer pandemic wave (01 June – 30 August 2009, case and media reporting) and winter pandemic wave (31 August 2009 – 14 February 2010, case and media reporting). HPA/Q-Surveillance cases were examined at different geographic scales and evaluated by Pearson's correlation coefficients. For each product category, correlation between residual sales and cases was assessed for the period 4 May 2009 to 09 November 2009. As a rise in retail sales might be expected to occur before an outbreak is detected through healthcare based surveillance cross correlation with weekly time lags was also performed.

Spatial correlation was performed to look for evidence of clustering of residual sales and influenza cases at different time points. This was performed using the 'spatial test' function in R statistical language, included in the GeoR package [24]: this calculates a test statistic by Monte Carlo permutation testing for spatial autocorrelation based on the use of variograms. For each product group, this test statistic was calculated for sub-regional residual sales. These spatial correlations were then examined as part of the weekly time series.

All data adjustment and analysis was performed using R statistical software, version 2.15.2. Statistical significance was set at 95%.

Results

During the declared pandemic period there were two peaks of estimated cases in the summer and winter seasons seen in both HPA/Q-Surveillance and flusurvey data (Figure 1). Media reporting was high in the early pandemic period (where there were relatively few cases in the UK) and during the summer wave but was less during the winter wave. Unadjusted national retail sales are shown in Figure 1 on a logarithmic scale.

There was a statistically significant positive correlation between thermometer and anti-viral product sales and national cases for the whole pandemic period (Table 2). When divided into summer and winter pandemic waves, the correlation was stronger in the summer wave than the winter wave. Children's cold and flu remedies were also positively correlated with national cases during the summer wave but not in the winter wave. Correlation between weekly residual sales and weekly media reporting was also performed (Table 2 and SA Table A1). Thermometer and anti-viral products were significantly positively correlated with media reporting for the whole pandemic period (Cor 0.477 (95% CI 0.171-0.699; 0711 (95% CI 0.495-0.844) respectively). No product group sales were significantly associated with media reporting in the early pandemic period though the strength of correlation was higher in the summer than the winter wave (Table 2).

At a regional level there was no significant correlation between estimated influenza cases and retail sales of adult 'cold and flu' remedies, cough remedies or tissues. There were weak but statistically significant correlations between sales of children's remedies and cases in six English regions and Wales (SA Table A3). Stronger positive correlations were seen between thermometer and cases and hand-gel sales and cases across all English regions and Wales (SA Table A3). No additional significant correlations were identified through cross-correlation analysis.

For peer review only

At the sub-regional level there was a significant positive correlation between thermometer and hand-gel sales and cases in England (69.9%, 109/156; 71.8%, 112/156 respectively) (Figure 2). Several sub-regions had a statistically significant positive correlation between cases and sales of adult 'cold and flu' remedies (3.2%, 5/156) and cough remedies (3.8%, 6/156); however, a greater number of sub-regions had a significant correlation between cases and children's remedy sales (35.6%, 55/156).

We found periods of significant spatial structure throughout the pandemic period for all sale products (SA Figure A3), particularly for tissue and anti-viral products sales which appear to have more sustained periods of spatial patterning that the other product types.

Discussion

We analysed non-prescription retail sales data for a major GB supermarket in comparison with cases of pandemic influenza within Great Britain to assess the utility of purchase data to inform and augment existing surveillance methods. We found a poor match between symptomatic remedies and cases at the national scale for both summer and winter waves of the pandemic. However, we found a significant association between children's remedies and cases for the summer wave at the national scale, and sub-regional scales, where we found significant association in 55 out of 156 PCTs. Significant positive correlations between cases and sales of thermometers and anti-viral hand gels and hand wash were seen at all spatial scales.

One concern about the use of retail sales as a surveillance tool is that it may be more easily influenced by factors other than symptomatic cases, such as heightened media coverage, and promotional activity by manufacturers, supermarkets and government, than other forms of reporting. The greatest press coverage occurred during the early pandemic period where there were relatively few cases of pandemic influenza in the GB. The lack of correlation between sales and this heightened coverage during this period suggests that 'panic buying' of symptomatic remedies or non-pharmacological groups in response to media reports did not occur. The lack of correlation between sales and media reports in the winter suggests that sales were more driven by cases than media reports as there was a similar level of cases in both the summer and winter periods.

The use of sales information for adult and child remedy products has been suggested as a useful augmentation to traditional surveillance mechanisms [8 13 14], but has not been tested within the GB. Previous studies in other counties suggest that national-scale data is uninformative [12], but more localised data can reflect surveyed influenza patterns [11 25]. Our results broadly support these observations. Some products may be more useful than others in their relative ability to reflect underlying disease incidence [26]. Our results suggest that children's remedies may better reflect community infection patterns than adult products. This may be due to children being at higher risk of infection with 2009 pandemic influenza than adults [27], being more likely to be symptomatic [28 29], or may reflect adult-parent differences in self-medication practices [30].

Sales of anti-viral products and thermometers were highly associated with both pandemic influenza cases and media and public interest measures, especially during the first 'summer' wave of the

For peer review only – http://bmjopen.bmj.com/site/about/guidelines.xhtml

pandemic. The use of anti-viral products and thermometers (for self-diagnosis) were recommended by UK government public health messaging during the early months of the pandemic and throughout the pandemic [31]. Cross sectional telephone surveys have generally reported low level of uptake of public health advice [32 33] but there is some evidence that this is a poor indicator of actual behaviour when more objective measures are used [34]. We believe our results are the first national-scale evidence that the public actively responded to these messages, at least through the purchasing of such products, and provides an alternative objective measure of public response to health advice.

There are several limitations to this study. The sales data used here are derived from the shopping purchases of a sample of shopping baskets, and only from purchases involving presentation of a loyalty card. The sales data are only sourced from one supermarket chain, and while that chain has one of the largest market shares nationally in the UK, many non prescription purchases are likely to be made in other outlets (such as dedicated pharmacies) which may better reflect community incidence of infection. The available sales data, while resolved to purchases made at an individual store level, was only available at a weekly time resolution preventing more finely resolved temporal analysis. Remedy products may be purchased for a variety of reasons other than to directly medicate against infection with influenza: they could be used for symptom alleviation for a range of other pathogen infections and conditions. We do not know if and how purchasing patterns reflect the use of the products themselves: individuals may use previously purchased products at the onset of new symptoms, only purchasing products when these expire, rather than buying new products to treat a new illness. We did not have access to surveillance data at PCT level for the full pandemic period, which would have been very valuable. Case data used in this analysis was not stratified by age; we were therefore unable to perform a more appropriate comparison of case data with adult and children products. Purchasing patterns made over a greater number of years and influenza seasons could have improved the seasonality estimation of purchasing behaviour.

The pandemic of 2009 was of a mild strain, which did not appear to generate a large volume of community cases which self-mediated using OTC remedies and which did not present to existing surveillance mechanisms. However, at particular spatial scales and in particular age-groups, or (we suggest) for more severe strains, retail sales may help augment existing surveillance mechanisms to provide a quantitative indication of care-seeking behaviour. However, there remain considerable uncertainties in the specific usage and self-medicating behaviour of individuals in relation to infection and purchasing of products: further investigation is required prior to the use of sales data for surveillance purposes.

Conclusions

Retail sales of over the counter symptom remedies at a national level are unlikely to be useful for the detection of cases. However, at more finely resolved spatial scales and in particular age-groups retail sales may help augment existing influenza surveillance and merit further study. Our study demonstrates that the retail sales patterns of particular product types, such as personal hygiene and self-diagnosis products, can be of value in assessing public responses to regional and national health messaging.

For peer review only

Contributors

ST performed the analysis and took the lead in writing; JMR conceived the study; all authors designed the analysis and commented on manuscript drafts. The authors are grateful to John Edmunds and Ellen Brooks Pollock for providing Flu Survey case estimates and to James Rubin and Susan Michie for providing aggregated media article counts. JMR would like to thank Ashleigh Jellicoe and Xu-Sheng Zhang for assistance in compiling newspaper and retail sales data.

Funding

ST acknowledges funding from the Wellcome Trust (097465/B/11/Z). JMR acknowledges support for this work from the Economic and Social Research Council and the Medical Research Council (grant RES-355-25-0019). PJW thanks the Medical Research Council for centre funding.

Competing Interests: None.

References

1. World Health Organisation. International Health Regulations 2nd Ed ed, 2005.
2. Ortiz JR SV, Uez OC, Oliva O, Bettels D, McCarron M, et al. Strategy to enhance influenza surveillance worldwide. Emerg Infect Dis [serial on the Internet] 2009;Available from http://wwwnc.cdc.gov/eid/article/15/8/08-1422.htm
3. Triple S Project. Assessment of syndromic surveillance in Europe. The Lancet 2011;378(9806):1833-34 doi: http://dx.doi.org/10.1016/S0140-6736(11)60834-9[published Online First: Epub Date]|.
4. Briand S, Mounts A, Chamberland M. Challenges of global surveillance during an influenza pandemic. Public Health 2011;125(5):247-56 doi: 10.1016/j.puhe.2010.12.007[published Online First: Epub Date]|.
5. Lipsitch M, Hayden FG, Cowling BJ, Leung GM. How to maintain surveillance for novel influenza A H1N1 when there are too many cases to count. The Lancet 2009;374(9696):1209-11 doi: 10.1016/s0140-6736(09)61377-5[published Online First: Epub Date]|.
6. Severi E, Heinsbroek E, Watson C, Catchpole M. Infectious disease surveillance for the London 2012 Olympic and Paralympic Games. Euro surveillance : bulletin europeen sur les maladies transmissibles = European communicable disease bulletin 2012;17(31)
7. Dailey L, Watkins RE, Plant AJ. Timeliness of data sources used for influenza surveillance. Journal of the American Medical Informatics Association : JAMIA 2007;14(5):626-31 doi: 10.1197/jamia.M2328[published Online First: Epub Date]|.
8. Wagner MM, Tsui FC, Espino J, et al. National Retail Data Monitor for public health surveillance. MMWR. Morbidity and mortality weekly report 2004;53 Suppl:40-2
9. Welliver RC, Cherry JD, Boyer KM, et al. Sales of nonprescription cold remedies: a unique method of influenza surveillance. Pediatric research 1979;13(9):1015-7
10. Davies GR, Finch RG. Sales of over-the-counter remedies as an early warning system for winter bed crises. Clinical microbiology and infection : the official publication of the European Society of Clinical Microbiology and Infectious Diseases 2003;9(8):858-63
11. Das D, Metzger K, Heffernan R, Balter S, Weiss D, Mostashari F. Monitoring over-the-counter medication sales for early detection of disease outbreaks--New York City. MMWR. Morbidity and mortality weekly report 2005;54 Suppl:41-6
12. Ohkusa Y, Shigematsu M, Taniguchi K, Okabe N. Experimental surveillance using data on sales of over-the-counter medications--Japan, November 2003-April 2004. MMWR. Morbidity and mortality weekly report 2005;54 Suppl:47-52

For peer review only

13. Socan M, Erculj V, Lajovic J. Early detection of influenza like illness through medication sales. Central European journal of public health 2012;20(2):156-62
14. Goldenberg A, Shmueli G, Caruana RA, Fienberg SE. Early statistical detection of anthrax outbreaks by tracking over-the-counter medication sales. Proceedings of the National Academy of Sciences of the United States of America 2002;99(8):5237-40 doi: 10.1073/pnas.042117499[published Online First: Epub Date]|.
15. Burkom HS, Elbert Y, Feldman A, Lin J. Role of data aggregation in biosurveillance detection strategies with applications from ESSENCE. MMWR. Morbidity and mortality weekly report 2004;53 Suppl:67-73
16. Proctor ME, Blair KA, Davis JP. Surveillance data for waterborne illness detection: an assessment following a massive waterborne outbreak of Cryptosporidium infection. Epidemiology & Infection 1998;120(01):43-54
17. Kirian ML, Weintraub JM. Prediction of gastrointestinal disease with over-the-counter diarrheal remedy sales records in the San Francisco Bay Area. BMC medical informatics and decision making 2010;10:39 doi: 10.1186/1472-6947-10-39[published Online First: Epub Date]|.
18. Public Health England. Influenza Surveillance in the United Kingdom. Secondary Influenza Surveillance in the United Kingdom 2013. http://www.hpa.org.uk/web/HPAweb&HPAwebStandard/HPAweb_C/1195733821514.
19. Brooks-Pollock E, Tilston N, Edmunds WJ, Eames KT. Using an online survey of healthcare-seeking behaviour to estimate the magnitude and severity of the 2009 H1N1v influenza epidemic in England. BMC infectious diseases 2011;11:68
20. HARCOURT SE, SMITH GE, ELLIOT AJ, et al. Use of a large general practice syndromic surveillance system to monitor the progress of the influenza A(H1N1) pandemic 2009 in the UK. Epidemiology & Infection 2012;140(01):100-05 doi: doi:10.1017/S095026881100046X[published Online First: Epub Date]|.
21. LexisNexis. Secondary 2011. www.http://nexis.co.uk/.
22. Meltwater. Secondary 2011. http://www.meltwater.com/.
23. Google Trends. Secondary 2011. http://www.google.com/trends/.
24. Riberio J, Diggle P. geoR: A package for geostatistical analysis. R-NEWS 2001;1(2)
25. Vergu E, Grais RF, Sarter H, et al. Medication sales and syndromic surveillance, France. Emerging infectious diseases 2006;12(3):416-21
26. Wallstrom GL, Hogan WR. Unsupervised clustering of over-the-counter healthcare products into product categories. Journal of biomedical informatics 2007;40(6):642-8 doi: 10.1016/j.jbi.2007.03.008[published Online First: Epub Date]|.
27. Miller E, Hoschler K, Hardelid P, Stanford E, Andrews N, Zambon M. Incidence of 2009 pandemic influenza A H1N1 infection in England: a cross-sectional serological study. The Lancet 2010;375(9720):1100-08
28. Dawood FS, Iuliano AD, Reed C, et al. Estimated global mortality associated with the first 12 months of 2009 pandemic influenza A H1N1 virus circulation: a modelling study. Lancet Infect Dis 2012;12(9):687-95 doi: 10.1016/s1473-3099(12)70121-4[published Online First: Epub Date]|.
29. Presanis AM, Pebody RG, Paterson BJ, et al. Changes in severity of 2009 pandemic A/H1N1 influenza in England: a Bayesian evidence synthesis. BMJ 2011;343:d5408 doi: 10.1136/bmj.d5408[published Online First: Epub Date]|.
30. Vernacchio L, Kelly JP, Kaufman DW, Mitchell AA. Cough and Cold Medication Use by US Children, 1999–2006: Results From the Slone Survey. Pediatrics 2008;122(2):e323-e29 doi: 10.1542/peds.2008-0498[published Online First: Epub Date]|.
31. NHS Scotland; NHS Wales; Department of Health SSaPS. Important information about swine flu—leaflet. Secondary Important information about swine flu—leaflet. 2009.

For peer review only

www.nhs.uk/news/2009/04April/Documents/Swine%20Flu%20Leaflet_Web%20Version.pdf.

32. Rubin GJ, Amlôt R, Page L, Wessely S. Public perceptions, anxiety, and behaviour change in relation to the swine flu outbreak: cross sectional telephone survey. BMJ 2009;339 doi: 10.1136/bmj.b2651[published Online First: Epub Date]|.

33. SteelFisher GK, Blendon RJ, Ward JRM, Rapoport R, Kahn EB, Kohl KS. Public response to the 2009 influenza A H1N1 pandemic: a polling study in five countries. The Lancet Infectious Diseases 2012;12(11):845-50

34. Fleischman DS, Webster GD, Judah G, de Barra M, Aunger R, Curtis VA. Sensor recorded changes in rates of hand washing with soap in response to the media reports of the H1N1 pandemic in Britain. BMJ Open 2011;1(2) doi: 10.1136/bmjopen-2011-000127[published Online First: Epub Date]|.

For peer review only

Table 1: Data Sources of Influenza Case Estimates, Media Reporting and Public Interest

Data	Description	Source	Dates	Ref
Flu Survey	GB National Case Estimates	Adjusted healthcare-based surveillance system.	01 June 2009 - 08 Feb 2010	[19]
HPA/Q Surveillance	Regional Case Estimates	General Practitioner symptomatic surveillance	04 May 2009 – 15 Nov 2009	[20]
LexisNexis	UK Media Coverage	UK newspaper headlines with reference to A/H1N1pdm and related terms	25 April 2009 – 27 Dec 2009	[21]
Meltwater	UK Media Coverage	UK newspaper headlines, radio and television news items with reference to A/H1N1pdm and related terms	06 April 2009 – 19 April 2010	[22]
Google Trends	UK Internet Searches	Internet searches from UK IP addresses with reference to A/H1N1pdm and related terms	06 April 2009 - 28 Dec 2009	[23]

For peer review only

Table 2: Correlation between Retail Sales, National Cases and Media Interest* * <0.05; ** <0.01 ***<0.001

	Whole Pandemic Period (19 April 2009–14 Feb 2010)		*Early Pandemic (19 April -31 May 2009)*		*Summer Wave (01 June – 30 Aug20 09)*		*Winter Wave (31 Aug 2009 – 14 Feb 2010)*	
	**Cor**	**95% CI**	**Cor**	**95% CI**	**Cor**	**95% CI**	**Cor**	**95% CI**
	**FluSurvey Case Estimates**							
**ADULT COLD & FLU REMEDIES**	0.116	-0.216 0.424	-	-	0.193	-0.401 0.672	0.149	-0.270 0.521
**CHILDRENS' COLD & FLU REMEDIES**	-0.023	-0.344 0.303	-	-	0.778**	0.396 0.930	0.010	-0.395 0.412
**COUGH REMEDIES**	0.374*	0.056 0.622	-	-	0.245	-0.353 0.702	0.396	-0.009 0.689
**THERMOMETERS**	0.445**	0.142 0.672	-	-	0.935***	0.792 0.981	0.796***	0.579 0.908
**ANTI-VIRAL PRODUCTS**	0.072	-0.258 0.387	-	-	0.671*	0.190 0.892	0.014	-0.392 0.415
**TISSUES**	0.051	-0.278 0.369	-	-	0.128	-0.455 0.634	-0.057	-0.450 0.354
	**Meltwater Reports**							
**ADULT COLD & FLU REMEDIES**	-0.256	-0.488 0.010	-0.379	-0.855 0.444	0.151	-0.436 0.648	-0.399	-0.691 0.005
**CHILDRENS' COLD & FLU REMEDIES**	0.171	-0.099 0.417	0.447	-0.376 0.876	0.576*	0.037 0.856	-0.427*	-0.708 -0.029
**COUGH REMEDIES**	-0.225	-0.462 0.043	-0.447	-0.876 0.376	0.249	-0.350 0.703	-0.129	-0.506 0.290
**THERMOMETERS**	0.364**	0.110 0.574	0.374	-0.449 0.854	0.772**	0.384 0.928	0.378	-0.030 0.678
**ANTI-VIRAL PRODUCTS**	0.458***	0.219 0.645	0.537	-0.270 0.901	0.516	-0.049 0.831	-0.119	-0.498 0.299
**TISSUES**	-0.288	-0.514 -0.025	0.386	-0.437 0.858	0.241	-0.358 0.699	-0.451	-0.723 -0.059
	**Google Searches**							
**ADULT COLD & FLU REMEDIES**	0.051	-0.269 0.360	-0.241	-0.808 0.559	0.258	-0.341 0.708	-0.214	-0.619 0.281
**CHILDRENS' COLD & FLU REMEDIES**	0.369*	0.060 0.613	0.452	-0.371 0.877	0.716**	0.273 0.909	-0.303	-0.674 0.191
**COUGH REMEDIES**	-0.050	-0.360 0.270	-0.318	-0.836 0.498	0.295	-0.306 0.728	-0.083	-0.529 0.399
**THERMOMETERS**	0.661***	0.437 0.808	0.212	-0.579 0.797	0.891***	0.669 0.967	0.570*	0.140 0.819
**ANTI-VIRAL PRODUCTS**	0.562***	0.299 0.745	0.346	-0.474 0.845	0.610*	0.089 0.869	0.038	-0.437 0.496
**TISSUES**	-0.063	-0.371 0.257	0.196	-0.590 0.791	0.296	-0.305 0.728	-0.034	-0.493 0.440

Figure 1. Top Panel: Weekly estimated cases of influenza shown are from English GP surveillance system (HPA/Q-surveillance) and UK wide estimates adjusted for changes in care seeking behavior (Flu Survey). Middle Panel: Weekly sales per 100,000 customers of six product groups from a national UK retailer. Bottom Panel: Scaled weekly estimates of UK media interest (number of relevant newspaper headlines (LexisNexis) or newspaper, radio and television articles (Meltwater)); UK public interest is represented by relative internet search volume from Google Search Trends.

Figure 2. Correlations between sales of 6 product categories and Influenza A H1N1/pdm cases during 2009. Points relate to a geographic region, size of the point and depth of colour is related to the strength of the correlation.

For peer review only

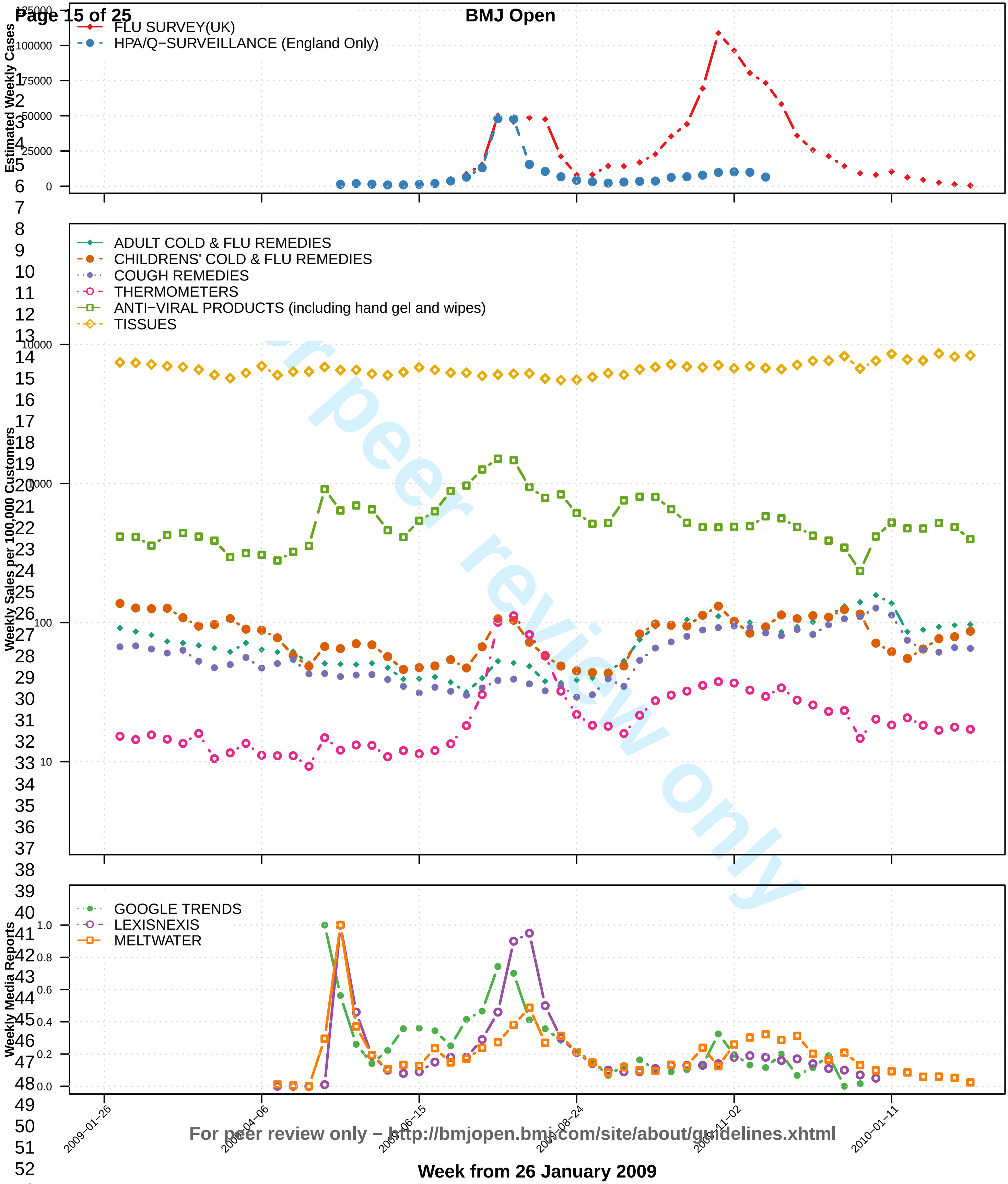
Estimated Weekly Cases
FLU SURVEY(UK)
HPA/Q−SURVEILLANCE (England Only)
125000
100000
75000
50000
25000
0
Weekly Sales per 100,000 Customers
ADULT COLD & FLU REMEDIES
CHILDRENS' COLD & FLU REMEDIES
COUGH REMEDIES
THERMOMETERS
ANTI−VIRAL PRODUCTS (including hand gel and wipes)
TISSUES
10000
1000
100
10
Weekly Media Reports
GOOGLE TRENDS
LEXISNEXIS
MELTWATER
1.0
0.8
0.6
0.4
0.2
0.0
2009−01−26
2009−04−06
2009−06−15
2009−08−24
2009−11−02
2010−01−11
Week from 26 January 2009

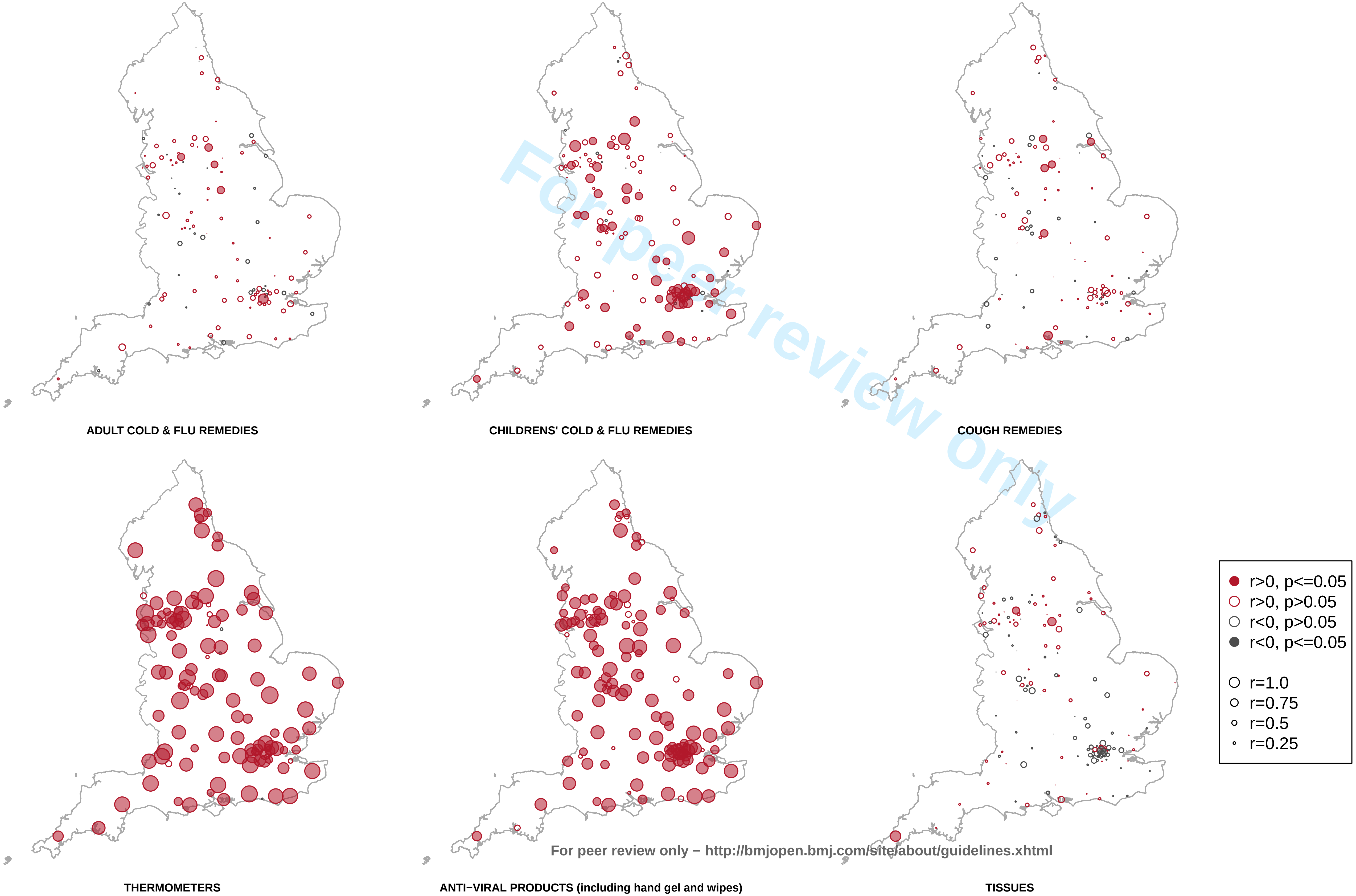

For peer review only

For peer review only − http://bmjopen.bmj.com/site/about/guidelines.xhtml

Table A1: Number of Sine/Cosine Pairs in Model Fit by Product Group and Region

	1 Sine/Cosine Pair	*2 Sine/Cosine Pair*	*3 Sine/Cosine Pair*	*4 Sine/Cosine Pair*	*Unable to Fit*
	**National**				
ADULT COLD & FLU REMEDIES	0	0	1	0	0
CHILDRENS' COLD & FLU REMEDIES	0	0	1	0	0
COUGH REMEDIES	0	0	1	0	0
THERMOMETERS	0	0	1	0	0
ANTI-VIRAL PRODUCTS	1	0	0	0	0
TISSUES	0	1	0	0	0
	**Regional**				
ADULT COLD & FLU REMEDIES	0	1	11	0	1
CHILDRENS' COLD & FLU REMEDIES	2	3	7	1	0
COUGH REMEDIES	0	0	12	0	1
THERMOMETERS	0	8	5	0	0
ANTI-VIRAL PRODUCTS	13	0	0	0	0
TISSUES	0	13	0	0	0
	**Subregional**				
ADULT COLD & FLU REMEDIES	39	39	37	6	30
CHILDRENS' COLD & FLU REMEDIES	88	37	24	1	1
COUGH REMEDIES	51	40	4	1	30
THERMOMETERS	108	14	4	1	24
ANTI-VIRAL PRODUCTS	143	6	2	0	0
TISSUES	41	105	4	1	0

For peer review only

Table A2: Correlation between Retail Sales, and Media Interest * <0.05; ** <0.01 ***<0.001

	Whole Pandemic Period (19 April 2009–14 Feb 2010)		*Early Pandemic (19 April -31 May 2009)*		*Summer Wave (01 June – 30 Aug2009)*		*Winter Wave (31 Aug 2009 – 14 Feb 2010)*	
	**Cor**	**95% CI**	**Cor**	**95% CI**	**Cor**	**95% CI**	**Cor**	**95% CI**
	**LexisNexis**							
**ADULT COLD & FLU REMEDIES**	0.169	-0.174 0.476	-0.872	-0.992 0.043	0.737**	0.313 0.916	0.171	-0.337 0.602
**CHILDRENS' COLD & FLU REMEDIES**	0.452**	0.140 0.682	-0.890*	-0.993 - 0.038	0.870***	0.612 0.960	0.101	-0.399 0.555
**COUGH REMEDIES**	-0.015	-0.347 0.319	-0.856	-0.990 0.107	0.760*	0.359 0.924	0.242	-0.270 0.647
**THERMOMETERS**	0.477**	0.171 0.699	0.536	-0.657 0.963	0.799**	0.444 0.937	0.559*	0.108 0.820
**ANTI-VIRAL PRODUCTS**	0.711***	0.495 0.844	0.786	-0.314 0.985	0.853***	0.569 0.955	0.212	-0.299 0.629
**TISSUES**	-0.149	-0.460 0.194	0.861	-0.089 0.991	-0.088	-0.610 0.486	0.141	-0.364 0.583

For peer review only

Table A2: Correlation between Retail Sales and Regional Cases * <0.05; ** <0.01 ***<0.001

	ADULT COLD & FLU REMEDIES		CHILDRENS' COLD & FLU REMEDIES		COUGH REMEDIES		THERMOMETERS		ANTI-VIRAL PRODUCTS (including hand gel and wipes)		TISSUES	
	Cor	95% CI	Cor	95% CI	Cor	95% CI	Cor	95% CI	Cor	95% CI	Cor	95% CI
**South Central**	0.211	-0.176 0.542	0.376*	0.003 0.657	0.128	-0.257 0.478	0.905***	0.803 0.955	0.673***	0.401 0.836	-0.012	-0.384 0.362
**East Of England**	0.049	-0.330 0.414	0.354	-0.022 0.643	0.034	-0.344 0.402	0.923***	0.838 0.964	0.778***	0.571 0.892	-0.082	-0.442 0.300
**London**	0.155	-0.232 0.499	0.553**	0.226 0.767	0.331	-0.048 0.627	0.860***	0.717 0.934	0.792***	0.595 0.900	-0.206	-0.538 0.181
**South East Coast**	0.163	-0.223 0.506	0.590**	0.278 0.789	0.077	-0.304 0.438	0.925***	0.842 0.965	0.768***	0.554 0.887	-0.052	-0.417 0.328
**South West**	0.105	-0.279 0.460	0.389*	0.019 0.666	0.003	-0.371 0.375	0.924***	0.840 0.964	0.658***	0.378 0.828	0.024	-0.352 0.394
**North West**	0.099	-0.284 0.456	0.189	-0.198 0.525	0.055	-0.325 0.419	0.934***	0.862 0.969	0.710***	0.459 0.856	0.082	-0.300 0.442
**East Midlands**	0.145	-0.242 0.491	0.323	-0.056 0.622	0.018	-0.357 0.388	0.926***	0.845 0.966	0.840***	0.680 0.924	0.046	-0.333 0.412
**West Midlands**	-0.089	-0.447 0.294	0.281	-0.103 0.592	0.030	-0.347 0.399	0.863***	0.723 0.935	0.795***	0.600 0.901	-0.025	-0.394 0.351
**Yorkshire And The Humber**	0.241	-0.145 0.563	0.437*	0.077 0.697	0.194	-0.193 0.529	0.926***	0.845 0.966	0.721***	0.477 0.862	0.147	-0.240 0.493
**Wales**	0.151	-0.235 0.496	0.418*	0.053 0.684	0.125	-0.260 0.476	0.944***	0.882 0.974	0.509**	0.168 0.741	0.216	-0.171 0.545
**North East**	0.268	-0.117 0.583	0.510**	0.169 0.742	0.227	-0.160 0.553	0.945***	0.884 0.975	0.728***	0.487 0.866	0.032	-0.345 0.400

For peer review only

Figure A1. For each geographic region and each product category a trend of weekly sales in 2008-2010 is available (grey line). A trend of seasonal sales is calculated from 2008 sales (trend line to left of the vertical black dotted line). The optimal number of sine/cosine pairs is selected using a maximised log likelihood method. From that a fitted line of the expected seasonal sales for that product group at that spatial resolution is generated (dashed red line). This seasonal trend acts as the 'expected baseline sales' for each corresponding week of 2009 and 2010. The residual sales are used within the analysis. National UK data is shown in this figure.

For peer review only

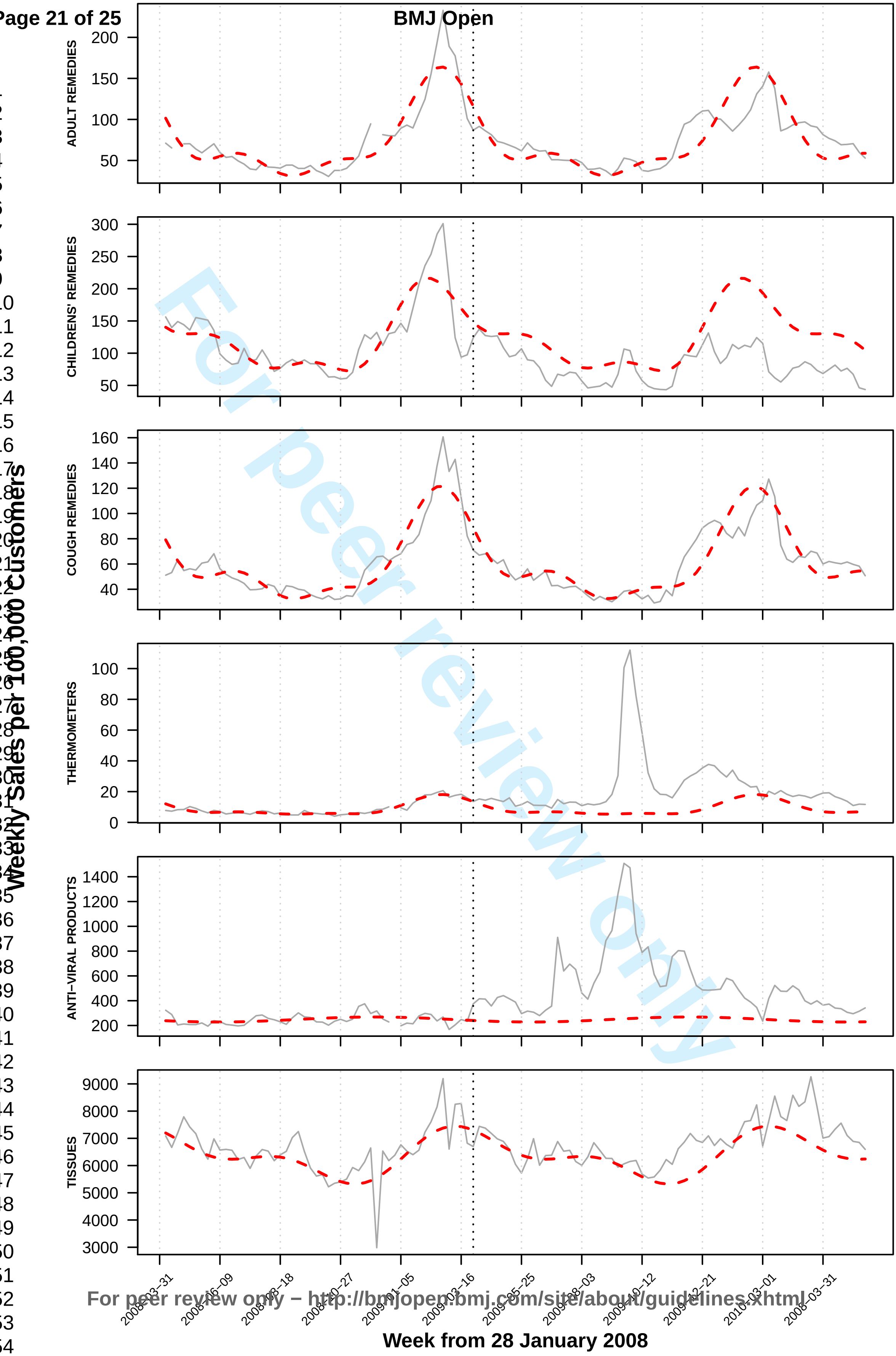

For peer review only

Figure A2. The optimal number of sine/cosine pairs for fitted line is calculated using a log likelihood method. This figure demonstrates the differences in optimal fitted lines (dashed red line) for different sub-regions based on 2008 sales of childrens' remedies (grey line to left of vertical black dotted line).

For peer review only

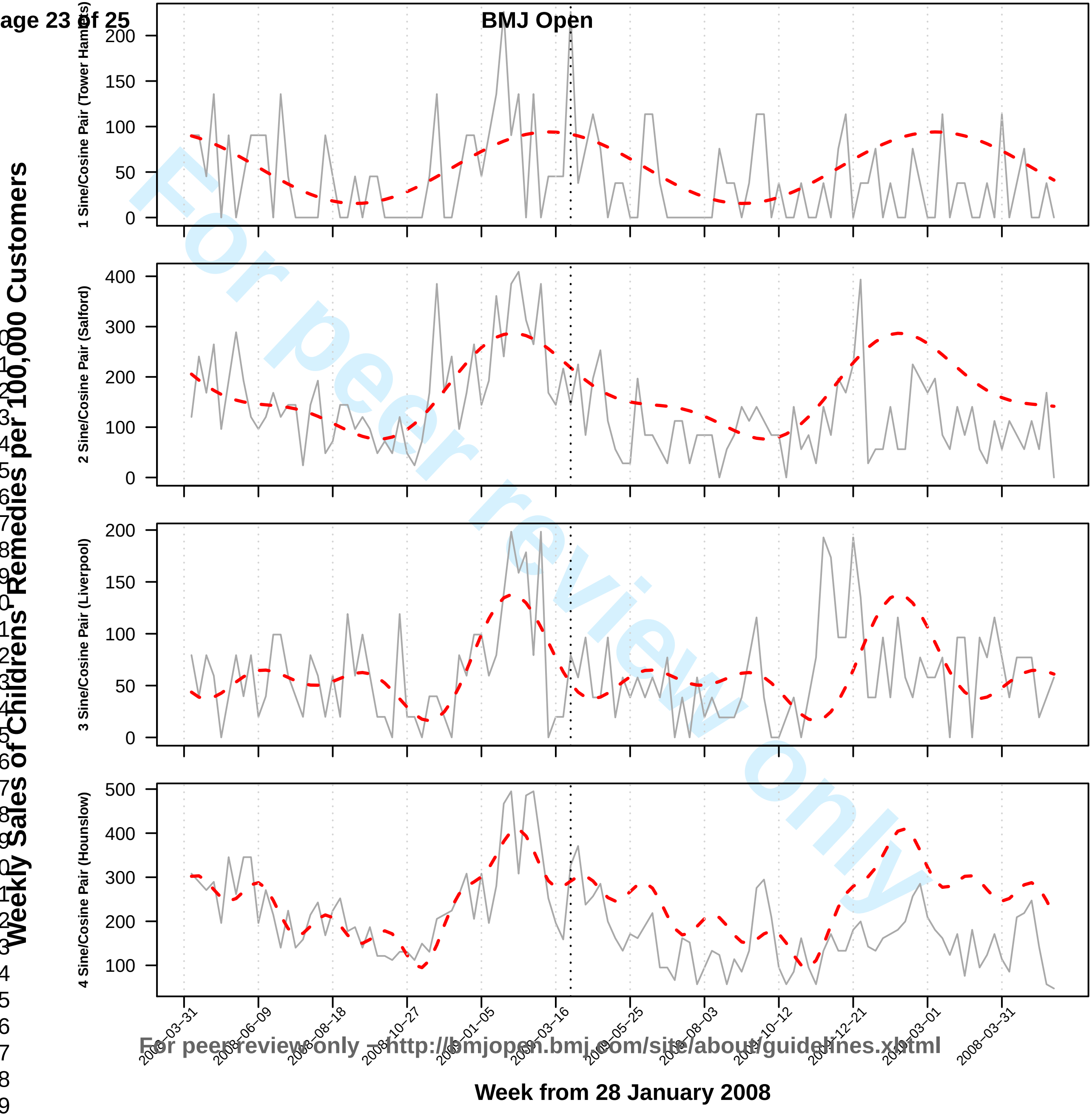

For peer review only

Figure A3. Test for spatial structure in sales of six product categories (whole time period) and cases of Influenza A H1N1 pdm (time period of available sub-regional case data is highlighted in red) at a sub-regional (PCT) level. This test was performed across 156 sub-regions for each week of the time period. A grey square indicates evidence of statistically significant spatial heterogeneity for the sales of that product group during that week. Darker grey indicates greater statistical significance. The coloured background regions indicate general specific periods of influenza activity (pink: early pandemic period, blue: summer pandemic wave; green: winter pandemic peak and seasonal peak in 2008.

For peer review only

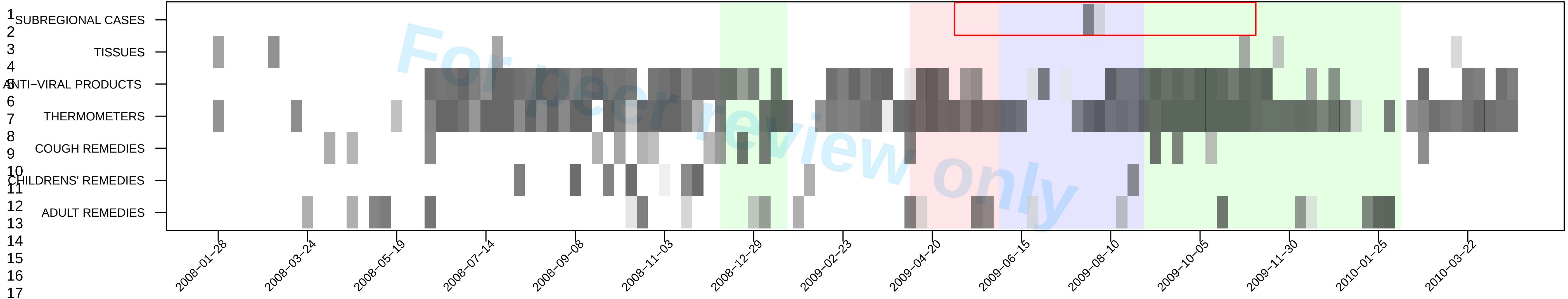

For peer review only − http://bmjopen.bmj.com/site/about/guidelines.xhtml

Week from 28 January 2008

BMJ Open

The spatio-temporal association of non-prescription retail sales with cases during the 2009 influenza pandemic in Great Britain.

Journal:	*BMJ Open*
Manuscript ID:	bmjopen-2014-004869.R1
Article Type:	Research
Date Submitted by the Author:	09-Apr-2014

Complete List of Authors:	Todd, Stacy; Liverpool School of Tropical Medicine, Clinical Sciences; Oxford University Clinical Research Unit,<br>Diggle, Peter; University of Liverpool, Institute of Infection and Global Health; University of Lancaster, Lancaster Medical School<br>White, Peter; Public Health England, Modelling and Economics Unit<br>Fearne, Andrew; University of Kent, Kent Business School<br>Read, Jonathan; University of Liverpool, Institute of Infection and Global Health

**Primary Subject Heading**:	Public health
Secondary Subject Heading:	Epidemiology, Infectious diseases
Keywords:	Influenza, Syndromic Surveillance, Outbreak

SCHOLARONE™ Manuscripts

For peer review only

The spatio-temporal association of non-prescription retail sales with cases during the 2009 influenza pandemic in Great Britain.

Stacy Todd[1,2], Peter J Diggle[3,4], Peter J White[5,6,7], Andrew Fearne[8], Jonathan M Read[3].

1. Dept of Clinical Sciences, Liverpool School of Tropical Medicine, UK
2. Oxford University Clinical Research Unit, Wellcome Trust Major Overseas Programme, Ho Chi Minh City, Vietnam
3. Institute of Infection and Global Health, University of Liverpool, UK
4. Lancaster Medical School, University of Lancaster, UK
5. MRC Centre for Outbreak Analysis and Modelling, Department of Infectious Disease Epidemiology, Imperial College School of Public Health, London, UK
6. NIHR Health Protection Research Unit in Modelling Methodology, Department of Infectious Disease Epidemiology, Imperial College School of Public Health, London,UK
7. Modelling and Economics Unit, Centre for Infectious Disease Surveillance and Control, Public Health England, London, UK
8. Kent Business School, University of Kent, UK

Corresponding Author:

Dr Jonathan M Read
Department of Epidemiology & Population Health,
Institute of Infection and Global Health,
Faculty of Health and Life Sciences,
University of Liverpool, Leahurst Campus,
Neston, South Wirral
United Kingdom CH64 7TE
E: jonread@liv.ac.uk
T: +44 151 794 6092
F: +44 151 794 6028

Key Words: Influenza; Syndromic Surveillance (MESH); Outbreak
Word Count: 2948

For peer review only

ABSTRACT

Objective: To assess whether retail sales of non-prescription products can be used for syndromic surveillance and whether it can detect influenza activity at different spatial scales. A secondary objective was to assess whether changes in purchasing behaviour related to public health advice or levels of media or public interest.

Setting: United Kingdom

Participants: National and regional influenza case estimates and retail sales from major British supermarket.

Outcome Measures: Weekly, seasonally-adjusted sales of over-the-counter symptom remedies and non-pharmaceutical products; recommended as part of the advice offered by public health agencies; were compared with weekly influenza case estimates. Comparisons were made at national and regional spatial resolutions. We also compared sales to national measures of contemporaneous media output and public interest (internet search volume) related to the pandemic.

Results: At a national scale there was no significant correlation between retail sales of symptom remedies and cases for the whole pandemic period in 2009. At the regional scale, a minority of regions showed statistically significant positive correlations between cases and sales of adult 'cold and flu' remedies and cough remedies [3.2%, 5/156, 3.8%, 6/156], but a greater number of regions showed a significant positive correlation between cases and symptomatic remedies for children [35.6%, 55/156]. Significant positive correlations between cases and sales of thermometers and anti-viral hand gels/wash were seen at both spatial scales [Cor 0.477 (95% CI 0.171-0.699); 0.711 (95% CI 0.495-0.844)]. We found no significant association between retail sales and media reporting or internet search volume.

Conclusion: This study provides evidence that the British public responded appropriately to health messaging about hygiene. Non-prescription retail sales at a national level are not useful for the detection of cases. However, at finer spatial scales, in particular age-groups, retail sales may help augment existing surveillance and merit further study.

For peer review only

ARTICLE SUMMARY

Strengths and limitations of the study

- This study is the first to examine associations between non-prescription retail sales and influenza cases at different spatial resolutions in a British setting and in particular it's potential as part of syndromic surveillance systems.
- The adjustment for seasonality in retail sales was fitted for each spatial resolution to attempt to capture regional differences which may exist.
- The inclusion of non-pharmaceutical products allowed for the first objective assessment of the response to government public health messaging.
- The main weakness of this study is that regional data was available only for England and for a portion of the 2009/2010 pandemic period.
- Increasing the years of sales data prior to the pandemic period would have provided a more robust estimate of sales trend in a typical year.

For peer review only

Introduction

Public health surveillance has traditionally relied on healthcare providers reporting selected notifiable conditions, usually with biological confirmation [1]. Although a key part of national and international health regulations, this system has well-recognised problems including delays in reporting and difficulty in identifying unusual activity [2]. Expansion of non-traditional surveillance methods has occurred over the last 2 decades, initially because of concerns regarding bioterrorism, and has now been adopted into routine public health systems in many countries. These methods (often referred to as Syndromic Surveillance Systems) offer a real-time or near-real-time collection of data from a variety of sources, ideally in an automated manner which allows early identification of the spread and impact of emerging public health threats and better estimates of incidence in seasonal outbreaks [3]. The 2009 influenza pandemic provided the motivation to adopt and appraise many of these methods [4 5]. In the UK many of the lessons learned during this time were subsequently adopted during the 2012 Olympics and Paralympics to identify any early infectious disease threat [6].

The surveillance of infectious diseases can be strongly affected by the care seeking behaviour of individuals [7]. As many individuals will self-medicate for mild illness, surveillance of non-prescription sales has been suggested as an adjunct to healthcare based surveillance to estimate the magnitude and dynamics of care seeking behaviour [8]. Its usefulness for surveillance of seasonal influenza [9-13] and other illnesses [14-17] has been examined for over 30 years with varying degrees of success. A major potential benefit of this type of surveillance system would be to provide more reliable estimates of incidence when the propensity to seek care is low or changeable, and to identify early-stage epidemics through unusual purchasing activity. Additionally, this type of surveillance may also provide more finely resolved spatio-temporal information on incidence. At present, retail sales are not used for syndromic surveillance in Great Britain.

The first two cases of influenza A H1N1 2009/pdm in the UK were confirmed on 27 April 2009 [18]. There was a considerable media response before this and through the summer months. In addition to this a major government campaign was launched ("Catch it, Kill it, Bin it"). This encouraged the use of clean tissues and regular hand washing/use of alcohol hand gel. A leaflet was distributed to every household in the UK on 5 May 2009 with this hygiene advice and also included information on accessing clinical advice [19]. As part of the response within England the National Pandemic Flu Service (NPFS) was established which provided online and telephone advice to individuals including access to anti-viral medication, this commenced on 23 July 2009 and operated until 10 February 2010. This was offered as an alternative to usual primary care services [20].

Here, we describe the temporal and spatial patterns of sales of over-the-counter flu and cold remedies and non-pharmaceutical products; recommended as part of the advice offered by public health agencies; sold by a major British supermarket. We compare these patterns to national, regional and sub-regional estimated cases of pandemic influenza during 2009 in Great Britain. We also compare the pattern of sales to national measures of media output and public interest (internet search volume) related to the pandemic during the same time period to assess their relationship to purchasing behaviour.

For peer review only

Methods

Data sources (Table 1)

The weekly estimates of influenza cases were obtained via the Health Protection Agency (HPA; now part of Public Health England) as part of their influenza surveillance systems [21]. UK-wide data was calculated via the FluSurvey project (www.flusurvey.org) which adjusted healthcare-based surveillance system outputs to account for changes in care-seeking behaviour during the pandemic; the study directly estimated the propensity of individuals to seek care (and therefore contribute to surveillance estimates) during the pandemic through an online survey of a community cohort and indirectly through NPFS consultation [22]. Regional case data was available through the HPA/Q-Surveillance network which monitors diagnoses of influenza-like-illness (ILI) recorded by general practitioners onto routine electronic systems and extracted on a daily and weekly basis [23]. Over 3,400 practices contribute to the system, which covers approximately 38% of the UK population; most of the practices are in England with fewer in Wales and Northern Ireland (NI). At the time of the 2009 pandemic no Scottish practices contributed to the system. The density of coverage allows reporting at country and regional levels. Regionally this corresponds to 10 English Strategic Health Authorities (SHAs) and 156 Primary Care Trusts (PCTs), which is the lowest unit of healthcare provision in England with an average population size of 350,000. The HPA/Q-Surveillance data was provided as daily counts of reported ILI cases in each PCT and including estimated population in each PCT for that day. This was aggregated to a weekly scale and converted to incidence as a rate of cases per 100,000 population. HPA/QSurveillance data was aggregated to 3 spatial resolutions; sub-regional, regional and country level (corresponding to PCT, SHA and England, Wales and NI respectively).

Two measures of media interest and one of public interest over time were compiled. Daily national newspaper article counts were compiled from the Lexis Nexis newspaper archive [24], counting articles with headlines containing “swine flu” or “h1n1”. The same search phrases were used to identify relevant articles on the Meltwater online database: this database includes newspaper, online, television and radio news articles and reporting [25]. Internet search trends were used as a proxy for public interest in the pandemic. This was derived from Google Insight search facility [26], and the daily relative volume of searches made where the search terms contained the terms “swine flu” or “h1n1” were collated.

Weekly unit sales of non-prescription retail products for a major national UK retailer were obtained for the period 28 January 2008 to 25 April 2010. These sales records were derived from a 10% sample of transactions where a loyalty card was presented at the point of purchase and were available at store level. Data on individual product sales were extracted from a master database and aggregated into six categories: Adult Cold and Flu Remedies; Children’s Cold and Flu Remedies; Cough Remedies; Thermometers; Anti-Viral Products (including hand gel and wipes); Tissues. Sales as a proportion of customer base were used instead of absolute sales to control for confounders such as changes in store hours in the period of the study or variation in market share between stores. Short shelf-life products were assumed to be indicative of total customer base. Sales were therefore adjusted in the first instance by dividing weekly total sales (for each category of product and spatial scale) by the average weekly sales of milk and bananas at the appropriate spatial scale (annual sales for 2008 and 2009 available).

For peer review only – http://bmjopen.bmj.com/site/about/guidelines.xhtml

The extreme seasonality associated with influenza (and subsequently symptomatic remedies) in temperate zones could introduce biases in the analysis. To adjust for this, an underlying seasonal trend in proportional sales was fitted to log-transformed retail sales data from the beginning of February 2008 to the end of January 2009. This was a pre-pandemic year, which we assumed to be typical of the seasonal trend in influenza incidence. A flexible way to represent a seasonal trend is through a sum of sine-cosine waves with frequencies corresponding to 1, 2, 3, etc cycles per year. For example, the model with 2 sine-cosine pairs is

$$ln(y_t + 1) = \alpha_0 + \alpha_1 \sin\left(\frac{2\pi t}{52}\right) + \beta_1 \cos\left(\frac{2\pi t}{52}\right) + \alpha_2 \sin\left(\frac{4\pi t}{52}\right) + \beta_2 \cos\left(\frac{4\pi t}{52}\right) + \varepsilon_t$$

where y_t is the retail sales data for each week of the year, t, during 2008, α and β terms are the regression coefficients for each sine and cosine function, and ε is an error term.

The model-fitting process was repeated for each product category at each spatial resolution. This resulted in between 1 and 4 sine-cosine pairs across the different product groups. In each case, the fitted seasonal model was used to derive weekly residuals for each week of the 2009 and 2010 data; these residuals, which are normalized with respect to normal non-pandemic seasonal sales, are used in the comparative analysis (Supplementary Appendix Table A1, Figure A1 and A2).

Pearson's correlation was performed between each product category, national UK cases and media reporting. Analysis was performed for the whole pandemic period as well as the early pandemic period (06 April- 01 June 2009, media reporting only), summer pandemic wave (01 June – 30 August 2009, case and media reporting) and winter pandemic wave (31 August 2009 – 14 February 2010, case and media reporting). HPA/Q-Surveillance cases were examined at different geographic scales and evaluated by Pearson's correlation coefficients. For each product category, correlation between residual sales and cases was assessed for the period 4 May 2009 to 09 November 2009. As a rise in retail sales might be expected to occur before an outbreak is detected through healthcare based surveillance cross correlation with weekly time lags was also performed.

Spatial correlation was performed to look for evidence of clustering of residual sales and influenza cases at different time points. This was performed using the 'spatial test' function in R statistical language, included in the GeoR package [27]: this calculates a test statistic by Monte Carlo permutation testing for spatial autocorrelation based on the use of variograms. For each product group, this test statistic was calculated for sub-regional residual sales. These spatial correlations were then examined as part of the weekly time series.

All data adjustment and analysis was performed using R statistical software, version 2.15.2. Statistical significance was set at 95%.

Results

During the declared pandemic period there were two peaks of estimated cases in the summer and winter seasons seen in national flusurvey data (Figure 1). HPA/QSurveillance data at a national scale did not show a winter peak. This is most likely due to the established presence of the NPFS service which triaged influenza like illness resulting in a reduced number of primary care consultations. Media reporting was high in the early pandemic period (where there were relatively few cases in the UK) and during the summer wave but was less during the winter wave. Unadjusted national retail sales are shown in Figure 1 on a logarithmic scale.

For peer review only

There was a statistically significant positive correlation between thermometer and anti-viral product sales and national cases for the whole pandemic period (Table 2). When divided into summer and winter pandemic waves, the correlation was stronger in the summer wave than the winter wave. Children's cold and flu remedies were also positively correlated with national cases during the summer wave but not in the winter wave. Correlation between weekly residual sales and weekly media reporting was also performed (Table 2 and SA Table A2). Thermometer and anti-viral products were significantly positively correlated with media reporting for the whole pandemic period (Cor 0.477 (95% CI 0.171-0.699); 0.711 (95% CI 0.495-0.844) respectively). No product group sales were significantly associated with media reporting in the early pandemic period though the strength of correlation was higher in the summer than the winter wave (Table 2 and SA Table A2).

At a regional level there was no significant correlation between estimated influenza cases and retail sales of adult 'cold and flu' remedies, cough remedies or tissues. There were weak but statistically significant correlations between sales of children's remedies and cases in six English regions and Wales (SA Table A3). Stronger positive correlations were seen between thermometer and cases and hand-gel sales and cases across all English regions and Wales (SA Table A3). No additional significant correlations were identified through cross-correlation analysis. The strongest correlation in cross-correlation testing was for no lag (0 weeks) for all comparisons.

At the sub-regional level there was a significant positive correlation between thermometer and hand-gel sales and cases in England (69.9%, 109/156; 71.8%, 112/156 respectively) (Figure 2). Several sub-regions had a statistically significant positive correlation between cases and sales of adult 'cold and flu' remedies (3.2%, 5/156) and cough remedies (3.8%, 6/156); however, a greater number of sub-regions had a significant correlation between cases and children's remedy sales (35.6%, 55/156).

We found periods of significant spatial structure throughout the pandemic period for all sale products (SA Figure A3), particularly for tissue and anti-viral products sales which appear to have more sustained periods of spatial patterning than the other product types.

Discussion

We analysed non-prescription retail sales data for a major UK supermarket in comparison with pandemic influenza syndromic case estimates within Great Britain to assess the utility of purchase data to reflect case estimates from existing surveillance methods. We found a poor match between symptomatic remedies and cases at the national scale for both summer and winter waves of the pandemic. However, we found a significant association between children's remedies and cases for the summer wave at the national scale, and sub-regional scales, where we found significant association in 55 out of 156 PCTs. Significant positive correlations between cases and sales of thermometers and anti-viral hand gels and hand wash were seen at all spatial scales.

One concern about the use of retail sales as a surveillance tool is that it may be more easily influenced by factors other than symptomatic cases, such as heightened media coverage, and promotional activity by manufacturers, supermarkets and government, than other forms of

For peer review only

reporting. The greatest press coverage occurred during the early pandemic period where there were relatively few cases of pandemic influenza in the UK. The lack of correlation between sales and this heightened coverage during this period suggests that 'panic buying' of symptomatic remedies or non-pharmacological groups in response to media reports did not occur. The lack of correlation between sales and media reports in the winter suggests that sales were more driven by cases than media reports as there was a similar level of cases in both the summer and winter periods.

The use of sales information for adult and child remedy products has been suggested as a useful augmentation to traditional surveillance mechanisms [8 13 14], but has not been tested within the UK. Previous studies have suggested that localised retail sales data is more reflective of surveyed influenza patterns than national level data [11 12 28] . Our results broadly support these observations. Some products may be more useful than others in their relative ability to reflect underlying disease incidence [29]. Our results suggest that children's remedies may better reflect community infection patterns than adult products. This may be due to children being at higher risk of infection with 2009 pandemic influenza than adults [30], being more likely to be symptomatic [31 32], or may reflect adult-parent differences in self-medication practices [33]. We find no evidence that retails sales may detect cases earlier than established surveillance systems, though our analysis is limited by data resolved at a weekly scale.

Sales of anti-viral products and thermometers were highly associated with both pandemic influenza cases and media and public interest measures, especially during the first 'summer' wave of the pandemic. This finding was not replicated in tissue sales and may reflect larger unit sales per 100,000 customers making signals harder to detect. The use of anti-viral products and thermometers (for self-diagnosis) were recommended by UK government public health messaging during the early months of the pandemic and throughout the pandemic [19]. Cross sectional telephone surveys have generally reported low level of uptake of public health advice [34 35] but there is some evidence that this is a poor indicator of actual behaviour when more objective measures are used [36]. We believe our results are the first national-scale evidence that the public actively responded to these messages, at least through the purchasing of such products, and provides an alternative objective measure of public response to health advice.

There are several limitations to this study. The sales data used here are derived from the shopping purchases of a sample of shopping baskets, and only from purchases involving presentation of a loyalty card. The sales data are only sourced from one supermarket chain, and while that chain has one of the largest market shares nationally in the UK, many non prescription purchases are likely to be made in other outlets (such as dedicated pharmacies) which may better reflect community incidence of infection. The available sales data, while resolved to purchases made at an individual store level, was only available at a weekly time resolution preventing more finely resolved temporal analysis. Sales of anti-pyretic medication not branded as 'cold and flu remedies' were excluded from our analysis because of concerns regarding the interpretation of signals from these products. Remedy products may be purchased for a variety of reasons other than to directly medicate against infection with influenza: they could be used for symptom alleviation for a range of other pathogen infections and conditions. We do not know if and how purchasing patterns reflect the use of the products themselves: individuals may use previously purchased products at the onset of new symptoms, only purchasing products when these expire, rather than buying new products to treat a

For peer review only

new illness. We did not have access to surveillance data at PCT level for the full pandemic period, which would have been very valuable. The case data to which we compared the retail sales information is largely based on diagnosis of influenza-like illness cases (syndromic illness) and not virologically confirmed cases. Case data used in this analysis was not stratified by age; we were therefore unable to perform a more appropriate comparison of case data with adult and children products. Purchasing patterns made over a greater number of years and influenza seasons could have improved the seasonality estimation of purchasing behaviour.

The pandemic of 2009 was of a mild strain, which did not appear to generate a large volume of community cases which self-medicated using OTC remedies and which did not present to existing surveillance mechanisms. However, at particular spatial scales and in particular age-groups, or (we suggest) for more severe strains, retail sales may help augment existing surveillance mechanisms to provide a quantitative indication of care-seeking behaviour. However, there remain considerable uncertainties in the specific usage and self-medicating behaviour of individuals in relation to infection and purchasing of products: further investigation is required prior to the use of sales data for surveillance purposes.

Conclusions

Retail sales of over the counter symptom remedies at a national level are unlikely to be useful for the detection of cases. However, at more finely resolved spatial scales and in particular age-groups retail sales may help augment existing influenza surveillance and merit further study. Our study demonstrates that the retail sales patterns of particular product types, such as personal hygiene and self-diagnosis products, can be of value in assessing public responses to regional and national health messaging.

For peer review only

Funding

ST acknowledges funding from the Wellcome Trust (097465/B/11/Z). JMR acknowledges support for this work from the Economic and Social Research Council (grant ES/K004255/1) and the Medical Research Council (grant RES-355-25-0019). PJW thanks the MRC for Centre funding, and also thanks the UK National Institute for Health Research Health Protection Research Unit (NIHR HPRU) in Modelling Methodology at Imperial College London in partnership with Public Health England (PHE) for funding..

The views expressed are those of the authors and not necessarily those of the NHS, the NIHR, the Department of Health, or Public Health England.

Contributors

ST performed the analysis and took the lead in writing; JMR conceived the study; all authors designed the analysis and commented on manuscript drafts. The authors are grateful to John Edmunds and Ellen Brooks Pollock for providing Flu Survey case estimates and to James Rubin and Susan Michie for providing aggregated media article counts. JMR would like to thank Ashleigh Jellicoe and Xu-Sheng Zhang for assistance in compiling newspaper and retail sales data.

Competing Interests: None.

Data Sharing Statement: Further information on PHE Influenza Surveillance results at www.hpa.org.uk.

For peer review only

References

1. World Health Organisation. International Health Regulations 2nd Ed ed, 2005.
2. Ortiz JR SV, Uez OC, Oliva O, et al. Strategy to enhance influenza surveillance worldwide. Emerg Infect Dis [serial on the Internet] 2009;Available from http://wwwnc.cdc.gov/eid/article/15/8/08-1422.htm
3. Triple SP. Assessment of syndromic surveillance in Europe. The Lancet 2011;378(9806):1833-34
4. Briand S, Mounts A, Chamberland M. Challenges of global surveillance during an influenza pandemic. Public Health 2011;125(5):247-56 doi: 10.1016/j.puhe.2010.12.007[published Online First: Epub Date]|.
5. Lipsitch M, Hayden FG, Cowling BJ, et al. How to maintain surveillance for novel influenza A H1N1 when there are too many cases to count. The Lancet 2009;374(9696):1209-11 doi: 10.1016/s0140-6736(09)61377-5[published Online First: Epub Date]|.
6. Severi E, Heinsbroek E, Watson C, et al. Infectious disease surveillance for the London 2012 Olympic and Paralympic Games. Euro surveillance : bulletin europeen sur les maladies transmissibles = European communicable disease bulletin 2012;17(31)
7. Dailey L, Watkins RE, Plant AJ. Timeliness of data sources used for influenza surveillance. Journal of the American Medical Informatics Association : JAMIA 2007;14(5):626-31 doi: 10.1197/jamia.M2328[published Online First: Epub Date]|.
8. Wagner MM, Tsui FC, Espino J, et al. National Retail Data Monitor for public health surveillance. MMWR. Morbidity and mortality weekly report 2004;53 Suppl:40-2
9. Welliver RC, Cherry JD, Boyer KM, et al. Sales of nonprescription cold remedies: a unique method of influenza surveillance. Pediatric research 1979;13(9):1015-7
10. Davies GR, Finch RG. Sales of over-the-counter remedies as an early warning system for winter bed crises. Clinical microbiology and infection : the official publication of the European Society of Clinical Microbiology and Infectious Diseases 2003;9(8):858-63
11. Das D, Metzger K, Heffernan R, et al. Monitoring over-the-counter medication sales for early detection of disease outbreaks--New York City. MMWR. Morbidity and mortality weekly report 2005;54 Suppl:41-6
12. Ohkusa Y, Shigematsu M, Taniguchi K, et al. Experimental surveillance using data on sales of over-the-counter medications--Japan, November 2003-April 2004. MMWR. Morbidity and mortality weekly report 2005;54 Suppl:47-52
13. Socan M, Erculj V, Lajovic J. Early detection of influenza like illness through medication sales. Central European journal of public health 2012;20(2):156-62
14. Goldenberg A, Shmueli G, Caruana RA, et al. Early statistical detection of anthrax outbreaks by tracking over-the-counter medication sales. Proceedings of the National Academy of Sciences of the United States of America 2002;99(8):5237-40 doi: 10.1073/pnas.042117499[published Online First: Epub Date]|.
15. Burkom HS, Elbert Y, Feldman A, et al. Role of data aggregation in biosurveillance detection strategies with applications from ESSENCE. MMWR. Morbidity and mortality weekly report 2004;53 Suppl:67-73
16. Proctor ME, Blair KA, Davis JP. Surveillance data for waterborne illness detection: an assessment following a massive waterborne outbreak of Cryptosporidium infection. Epidemiology & Infection 1998;120(01):43-54
17. Kirian ML, Weintraub JM. Prediction of gastrointestinal disease with over-the-counter diarrheal remedy sales records in the San Francisco Bay Area. BMC medical informatics and decision making 2010;10:39 doi: 10.1186/1472-6947-10-39[published Online First: Epub Date]|.
18. Health Protection Agency. Epidemiological report of pandemic (H1N1) 2009 in UK. http://www.hpa.org.uk/Publications/InfectiousDiseases/Influenza/1010EpidemiologicalreportofpandemicH1N12009inUK/, 2010.

For peer review only

19. NHS Scotland; NHS Wales; Department of Health SSaPS. Important information about swine flu—leaflet. Last Update 04 April 2014. www.nhs.uk/news/2009/04April/Documents/Swine%20Flu%20Leaflet_Web%20Version.pdf.
20. Rutter P, Mytton O, Ellis B, et al. Access to the NHS by telephone and Internet during an influenza pandemic: an observational study. BMJ Open 2014;4(2) doi: 10.1136/bmjopen-2013-004174[published Online First: Epub Date]|.
21. Public Health England. Influenza Surveillance in the United Kingdom. Last Update. http://www.hpa.org.uk/web/HPAweb&HPAwebStandard/HPAweb_C/1195733821514.
22. Brooks-Pollock E, Tilston N, Edmunds WJ, et al. Using an online survey of healthcare-seeking behaviour to estimate the magnitude and severity of the 2009 H1N1v influenza epidemic in England. BMC infectious diseases 2011;11:68
23. HarcourtT SE, Smith GE, Elliot AJ, et al. Use of a large general practice syndromic surveillance system to monitor the progress of the influenza A(H1N1) pandemic 2009 in the UK. Epidemiology & Infection 2012;140(01):100-05 doi: doi:10.1017/S095026881100046X[published Online First: Epub Date]|.
24. LexisNexis. Nexis | Home. Last Update 16 January 2014. http://www.nexis.co.uk/.
25. Meltwater. Meltwater - Online Media Monitoring - Public Relations Software - Social Marketing Software. Last Update 16 January 2014. http://www.meltwater.com/.
26. Google. Google Trends. Last Update 16 January 2014. http://www.google.com/trends/.
27. Riberio J, Diggle P. geoR: A package for geostatistical analysis. R-NEWS 2001;1(2)
28. Vergu E, Grais RF, Sarter H, et al. Medication sales and syndromic surveillance, France. Emerging infectious diseases 2006;12(3):416-21
29. Wallstrom GL, Hogan WR. Unsupervised clustering of over-the-counter healthcare products into product categories. Journal of biomedical informatics 2007;40(6):642-8 doi: 10.1016/j.jbi.2007.03.008[published Online First: Epub Date]|.
30. Miller E, Hoschler K, Hardelid P, et al. Incidence of 2009 pandemic influenza A H1N1 infection in England: a cross-sectional serological study. The Lancet 2010;375(9720):1100-08
31. Dawood FS, Iuliano AD, Reed C, et al. Estimated global mortality associated with the first 12 months of 2009 pandemic influenza A H1N1 virus circulation: a modelling study. Lancet Infect Dis 2012;12(9):687-95 doi: 10.1016/s1473-3099(12)70121-4[published Online First: Epub Date]|.
32. Presanis AM, Pebody RG, Paterson BJ, et al. Changes in severity of 2009 pandemic A/H1N1 influenza in England: a Bayesian evidence synthesis. BMJ 2011;343:d5408 doi: 10.1136/bmj.d5408[published Online First: Epub Date]|.
33. Vernacchio L, Kelly JP, Kaufman DW, et al. Cough and Cold Medication Use by US Children, 1999–2006: Results From the Slone Survey. Pediatrics 2008;122(2):e323-e29 doi: 10.1542/peds.2008-0498[published Online First: Epub Date]|.
34. Rubin GJ, Amlôt R, Page L, et al. Public perceptions, anxiety, and behaviour change in relation to the swine flu outbreak: cross sectional telephone survey. BMJ 2009;339 doi: 10.1136/bmj.b2651[published Online First: Epub Date]|.
35. SteelFisher GK, Blendon RJ, Ward JRM, et al. Public response to the 2009 influenza A H1N1 pandemic: a polling study in five countries. The Lancet Infectious Diseases 2012;12(11):845-50
36. Fleischman DS, Webster GD, Judah G, et al. Sensor recorded changes in rates of hand washing with soap in response to the media reports of the H1N1 pandemic in Britain. BMJ Open 2011;1(2) doi: 10.1136/bmjopen-2011-000127[published Online First: Epub Date]|.

For peer review only

Table 1: Data Sources of Influenza Case Estimates, Media Reporting and Public Interest

Data	Description	Source	Dates	Ref
Flu Survey	UK National Case Estimates	Adjusted healthcare-based surveillance system.	01 June 2009 - 08 Feb 2010	[22]
HPA/Q Surveillance	Regional Case Estimates	General Practitioner symptomatic surveillance	04 May 2009 – 15 Nov 2009	[23]
LexisNexis	UK Media Coverage	UK newspaper headlines with reference to A/H1N1pdm and related terms	25 April 2009 – 27 Dec 2009	[24]
Meltwater	UK Media Coverage	UK newspaper headlines, radio and television news items with reference to A/H1N1pdm and related terms	06 April 2009 – 19 April 2010	[25]
Google Trends	UK Internet Searches	Internet searches from UK IP addresses with reference to A/H1N1pdm and related terms	06 April 2009 - 28 Dec 2009	[26]

For peer review only

Table 2: Correlation between Retail Sales, National Cases and Media Interest* * <0.05; ** <0.01 ***<0.001

	Whole Pandemic Period (19 April 2009–14 Feb 2010)		*Early Pandemic (19 April -31 May 2009)*		*Summer Wave (01 June – 30 Aug20 09)*		*Winter Wave (31 Aug 2009 – 14 Feb 2010)*	
	**Cor**	**95% CI**	**Cor**	**95% CI**	**Cor**	**95% CI**	**Cor**	**95% CI**
	**FluSurvey Case Estimates**							
**ADULT COLD & FLU REMEDIES**	0.116	-0.216 0.424	-	-	0.193	-0.401 0.672	0.149	-0.270 0.521
**CHILDRENS' COLD & FLU REMEDIES**	-0.023	-0.344 0.303	-	-	0.778**	0.396 0.930	0.010	-0.395 0.412
**COUGH REMEDIES**	0.374*	0.056 0.622	-	-	0.245	-0.353 0.702	0.396	-0.009 0.689
**THERMOMETERS**	0.445**	0.142 0.672	-	-	0.935***	0.792 0.981	0.796***	0.579 0.908
**ANTI-VIRAL PRODUCTS**	0.072	-0.258 0.387	-	-	0.671*	0.190 0.892	0.014	-0.392 0.415
**TISSUES**	0.051	-0.278 0.369	-	-	0.128	-0.455 0.634	-0.057	-0.450 0.354
	**Meltwater Reports**							
**ADULT COLD & FLU REMEDIES**	-0.256	-0.488 0.010	-0.379	-0.855 0.444	0.151	-0.436 0.648	-0.399	-0.691 0.005
**CHILDRENS' COLD & FLU REMEDIES**	0.171	-0.099 0.417	0.447	-0.376 0.876	0.576*	0.037 0.856	-0.427*	-0.708 -0.029
**COUGH REMEDIES**	-0.225	-0.462 0.043	-0.447	-0.876 0.376	0.249	-0.350 0.703	-0.129	-0.506 0.290
**THERMOMETERS**	0.364**	0.110 0.574	0.374	-0.449 0.854	0.772**	0.384 0.928	0.378	-0.030 0.678
**ANTI-VIRAL PRODUCTS**	0.458***	0.219 0.645	0.537	-0.270 0.901	0.516	-0.049 0.831	-0.119	-0.498 0.299
**TISSUES**	-0.288	-0.514 -0.025	0.386	-0.437 0.858	0.241	-0.358 0.699	-0.451	-0.723 -0.059
	**Google Searches**							
**ADULT COLD & FLU REMEDIES**	0.051	-0.269 0.360	-0.241	-0.808 0.559	0.258	-0.341 0.708	-0.214	-0.619 0.281
**CHILDRENS' COLD & FLU REMEDIES**	0.369*	0.060 0.613	0.452	-0.371 0.877	0.716**	0.273 0.909	-0.303	-0.674 0.191
**COUGH REMEDIES**	-0.050	-0.360 0.270	-0.318	-0.836 0.498	0.295	-0.306 0.728	-0.083	-0.529 0.399
**THERMOMETERS**	0.661***	0.437 0.808	0.212	-0.579 0.797	0.891***	0.669 0.967	0.570*	0.140 0.819
**ANTI-VIRAL PRODUCTS**	0.562***	0.299 0.745	0.346	-0.474 0.845	0.610*	0.089 0.869	0.038	-0.437 0.496
**TISSUES**	-0.063	-0.371 0.257	0.196	-0.590 0.791	0.296	-0.305 0.728	-0.034	-0.493 0.440

Fiure Legends:

Figure 1. Top Panel: Weekly estimated cases of influenza shown are from English GP surveillance system (HPA/Q-surveillance) and UK wide estimates adjusted for changes in care seeking behaviour (Flu Survey). Middle Panel: Weekly sales per 100,000 customers of six product groups from a national UK retailer. Bottom Panel: Scaled weekly estimates of UK media interest (number of relevant newspaper headlines (LexisNexis) or newspaper, radio and television articles (Meltwater)); UK public interest is represented by relative internet search volume from Google Search Trends.

Figure 2. Correlations between sales of 6 product categories and Influenza A H1N1/pdm cases during 2009. Points relate to a geographic region, size of the point and depth of colour is related to the strength of the correlation.

Supplementary Figures:

Figure A1. For each geographic region and each product category a trend of weekly sales in 2008-2010 is available (grey line). A trend of seasonal sales is calculated from 2008 sales (trend line to left of the vertical black dotted line). The optimal number of sine/cosine pairs is selected using a maximised log likelihood method. From that a fitted line of the expected seasonal sales for that product group at that spatial resolution is generated (dashed red line). This seasonal trend acts as the 'expected baseline sales' for each corresponding week of 2009 and 2010. The residual sales are used within the analysis. National UK data is shown in this figure.

Figure A2. The optimal number of sine/cosine pairs for fitted line is calculated using a log likelihood method. This figure demonstrates the differences in optimal fitted lines (dashed red line) for different sub-regions based on 2008 sales of childrens' remedies (grey line to left of vertical black dotted line).

Figure A3. Test for spatial structure in sales of six product categories (whole time period) and cases of Influenza A H1N1 pdm (time period of available sub-regional case data is highlighted in red) at a sub-regional (PCT) level. This test was performed across 156 sub-regions for each week of the time period. A grey square indicates evidence of statistically significant spatial heterogeneity for the sales of that product group during that week. Darker grey indicates greater statistical significance. The coloured background regions indicate general specific periods of influenza activity (pink: early pandemic period, blue: summer pandemic wave; green: winter pandemic peak and seasonal peak in 2008.

Supplementary tables

Table A1: Number of Sine/Cosine Pairs in Model Fit by Product Group and Region

Table A2: Correlation between Retail Sales, and Media Interest

Table A3: Correlation between Retail Sales and Regional Cases

For peer review only

The spatio-temporal association of non-prescription retail sales with cases during the 2009 influenza pandemic in Great Britain.

Stacy Todd[1,2], Peter J Diggle[3,4], Peter J White[5,6,7], Andrew ~~Fearne~~[7]Fearne[8], Jonathan M Read[3].

1. Dept of Clinical Sciences, Liverpool School of Tropical Medicine, UK
2. Oxford University Clinical Research Unit, Wellcome Trust Major Overseas Programme, Ho Chi Minh City, Vietnam
3. Institute of Infection and Global Health, University of Liverpool, UK
4. Lancaster Medical School, University of Lancaster, UK
5. MRC Centre for Outbreak Analysis and Modelling, Department of Infectious Disease Epidemiology, Imperial College School of Public Health, London, UK
6. NIHR Health Protection Research Unit in Modelling Methodology, Department of Infectious Disease Epidemiology, Imperial College School of Public Health, London,UK
7. Modelling and Economics Unit, Centre for Infectious Disease Surveillance and Control, Public Health England, London, UK
8. Kent Business School, University of Kent, UK

~~5. School of Public Health, Imperial College, UK~~
~~6. Modelling and Economics Unit , Public Health England, UK~~
~~Kent Business School, University of Kent, UK~~

Corresponding Author:

Dr Jonathan M Read
Department of Epidemiology & Population Health,
Institute of Infection and Global Health,
Faculty of Health and Life Sciences,
University of Liverpool, Leahurst Campus,
Neston, South Wirral
United Kingdom CH64 7TE
E: jonread@liv.ac.uk
T: +44 151 794 6092
F: +44 151 794 6028

Key Words: Influenza; Syndromic Surveillance (MESH); Outbreak
Word Count: ~~2645~~2948

For peer review only

ABSTRACT

~~**Background**: As many individuals will self medicate for mild influenza-like illness, surveillance of non-prescription purchases may be an important adjunct to healthcare-based surveillance in early assessment of the severity of a novel influenza strain or other pathogen. Its usefulness as a marker of seasonal influenza has been investigated for over 30 years with varying degrees of success.~~

Objective: ~~The aim of this paper was to compare spatio-temporal patterns of retail sales, influenza cases during the 2009 influenza pandemic.~~To assess whether retail sales of non-prescription products can be used for syndromic surveillance and whether it can detect influenza activity at different spatial scales. A secondary objective was to assess whether changes in purchasing behaviour related to public health advice or levels of media or public interest.

Setting: United Kingdom

Participants: National and regional influenza case estimates and retail sales from major British supermarket.

~~Methods~~Outcome Measures: Weekly, seasonally-adjusted sales ~~by a major British supermarket~~ of over-the-counter symptom remedies and non-pharmaceutical products; recommended as part of the advice offered by public health agencies; were compared with weekly influenza case estimates. Comparisons were made at national and regional spatial resolutions. We also compared sales to national measures of contemporaneous media output and public interest (internet search volume) related to the pandemic.

Results: At a national scale there was no significant correlation between retail sales of symptom remedies and cases for the whole pandemic period in 2009. At the regional scale, a minority of regions showed statistically significant positive correlations between cases and sales of adult 'cold and flu' remedies and cough remedies [3.2%, 5/156, 3.8%, 6/156], but a greater number of regions showed a significant positive correlation between cases and symptomatic remedies for children [35.6%, 55/156]. Significant positive correlations between cases and sales of thermometers and anti-viral hand gels/wash were seen at both spatial scales [Cor 0.477 (95% CI 0.171-0.699); 0.711 (95% CI 0.495-0.844)]. We found no significant association between retail sales and media reporting or internet search volume.

Conclusion: This study provides evidence that the British public responded appropriately to health messaging about hygiene. Non-prescription ~~R~~retail sales at a national level are not useful for the detection of cases. However, at finer spatial scales, in particular age-groups, retail sales may help augment existing surveillance and merit further study.

Formatted: Font: Bold

For peer review only

ARTICLE SUMMARY

Strengths and limitations of the study

- This study is the first to examine associations between non-prescription retail sales and influenza cases at different spatial resolutions in a British setting and in particular it's potential as part of syndromic surveillance systems.
- The adjustment for seasonality in retail sales was fitted for each spatial resolution to attempt to capture regional differences which may exist.
- The inclusion of non-pharmaceutical products allowed for the first objective assessment of the response to government public health messaging.
- The main weakness of this study is that regional data was available only for England and for a portion of the 2009/2010 pandemic period.
- Increasing the years of sales data prior to the pandemic period would have provided a more robust estimate of sales trend in a typical year.

For peer review only

Introduction

Public health surveillance has traditionally relied on healthcare providers reporting selected notifiable conditions, usually with biological confirmation [1]. Although a key part of national and international health regulations, this system has well-recognised problems including delays in reporting and difficulty in identifying unusual activity [2]. Expansion of non-traditional surveillance methods has occurred over the last 2 decades, initially because of concerns regarding bioterrorism, and has now been adopted into routine public health systems in many countries. These methods (often referred to as Syndromic Surveillance Systems) offer a real-time or near-real-time collection of data from a variety of sources, ideally in an automated manner which allows early identification of the spread and impact of potential emerging public health threats and better estimates of incidence in seasonal outbreaks [3]. The 2009 influenza pandemic provided the motivation to adopt and appraise many of these methods [4 5]. In the UK many of the lessons learned during this time were subsequently adopted during the 2012 Olympics and Paralympics to identify any early infectious disease threat [6].

The surveillance of infectious diseases can be strongly affected by the care seeking behaviour of individuals [7]. As many individuals will self-medicate for mild illness, surveillance of non-prescription sales has been suggested as an adjunct to healthcare based surveillance to estimate the magnitude and dynamics of care seeking behaviour [8]. Its usefulness for surveillance of seasonal influenza [9-13] and other illnesses [14-17] has been examined for over 30 years with varying degrees of success. A major potential benefit of this type of surveillance system would be to provide more reliable estimates of incidence when the propensity to seek care is low or changeable, and to identify early-stage epidemics through unusual purchasing activity. Additionally, this type of surveillance may also provide more finely resolved spatio-temporal information on incidence. At present, retail sales are not used for syndromic surveillance in Great Britain.

The first two cases of influenza A H1N1 2009/pdm in the UK were confirmed on 27 April 2009 [18]. There was a considerable media response before this and through the summer months. In addition to this a major government campaign was launched ("Catch it, Kill it, Bin it"). This encouraged the use of clean tissues and regular hand washing/use of alcohol hand gel. A leaflet was distributed to every household in the UK on 5 May 2009 with this hygiene advice and also included information on accessing clinical advice [19]. As part of the response within England the National Pandemic Flu Service (NPFS) was established which provided online and telephone advice to individuals including access to anti-viral medication, this commenced on 23 July 2009 and operated until 10 February 2010. This was offered as an alternative to usual primary care services [20].

Here, we describe the temporal and spatial patterns of sales of over-the-counter flu and cold remedies and non-pharmaceutical products; recommended as part of the advice offered by public health agencies; sold by a major British supermarket. , andWe compare these patterns to national, regional and sub-regional estimated cases of pandemic influenza during 2009 in Great Britain. We also compare the pattern of sales to national measures of media output and public interest (internet search volume) related to the pandemic during the same time period to assess their relationship to purchasing behaviour.

For peer review only

Methods

Data sources (Table 1)

The weekly estimates of influenza cases were obtained via the Health Protection Agency (HPA; now part of Public Health England) as part of their influenza surveillance systems [21]. UK-wide data was calculated via the FluSurvey project (www.flusurvey.org) which adjusted healthcare-based surveillance system outputss to account for changes in care-seeking behaviour during the pandemic;, as determined though an online survey of a community cohort the study directly estimated the propensity of individuals to seek care (and therefore contribute to surveillance estimates) during the pandemic through an online survey of a community cohort and indirectly through NPFS consultation [22]. Regional case data was available through the HPA/Q-Surveillance network which monitors diagnoses of influenza-like-illness (ILI) recorded by general practitioners onto routine electronic systems and extracted on a daily and weekly basis [23]. Over 3,400 practices contribute to the system, which covers approximately 38% of the UK population; most of the practices are in England with fewer in Wales and Northern IslandIreland (NI). At the time of the 2009 pandemic no Scottish practices contributed to the system. The density of coverage allows reporting at country and regional levels. Regionally this corresponds to 10 English Strategic Health Authorities (SHAs) and 156 Primary Care Trusts (PCTs), which is the lowest unit of healthcare provision in England with an average population size of 350,000. The HPA/Q-Surveillance data was provided as daily counts of reported ILI cases in each PCT and including estimated population in each PCT for that day. This was aggregated to a weekly scale and converted to incidence as a rate of cases per 100,000 population. HPA/QSurveillance data was aggregated to 3 spatial resolutions; sub-regional, regional and country level (corresponding to PCT, SHA and England, Wales and NI respectively).

Two measures of media interest and one of public interest over time were compiled. Daily national newspaper article counts were compiled from the Lexis Nexis newspaper archive [24], counting articles with headlines containing “swine flu” or “h1n1”. The same search phrases were used to identify relevant articles on the Meltwater online database: this database includes newspaper, online, television and radio news articles and reporting [25]. Internet search trends were used as a proxy for public interest in the pandemic. This was derived from Google Insight search facility [26], and the daily relative volume of searches made where the search terms contained the terms “swine flu” or “h1n1” were collated.

Weekly unit sales of non-prescription retail products for a major national UK retailer were obtained for the period 28 January 2008 to 25 April 2010. These sales records were derived from a 10% sample of transactions where a loyalty card was presented at the point of purchase and were available at store level. Data on individual product sales were extracted from a master database and aggregated into six categories: Adult Cold and Flu Remedies; Children’s Cold and Flu Remedies; Cough Remedies; Thermometers; Anti-Viral Products (including hand gel and wipes); Tissues. Sales as a proportion of customer base were used instead of absolute sales to control for confounders such as changes in store hours in the period of the study or variation in market share between stores. Short shelf-life products were assumed to be indicative of total customer base. Sales were therefore adjusted in the first instance by dividing weekly total sales (for each category of product and spatial scale) by the average weekly sales of milk and bananas at the appropriate spatial scale (annual sales for 2008 and 2009 available).

For peer review only

The extreme seasonality associated with influenza (and subsequently symptomatic remedies) in temperate zones could introduce biases in the analysis. To adjust for this, an underlying seasonal trend in proportional sales was fitted to log-transformed retail sales data from the beginning of February 2008 to the end of January 2009. This was a pre-pandemic year, which we assumed to be typical of the seasonal trend in influenza incidence. A flexible way to represent a seasonal trend is through a sum of sine-cosine waves with frequencies corresponding to 1, 2, 3, etc cycles per year. For example, the model with 2 sine-cosine pairs is

$$ln(y_t + 1) = \alpha_0 + \alpha_1 \sin\left(\frac{2\pi t}{52}\right) + \beta_1 \cos\left(\frac{2\pi t}{52}\right) + \alpha_2 \sin\left(\frac{4\pi t}{52}\right) + \beta_2 \cos\left(\frac{4\pi t}{52}\right) + \varepsilon_t$$

where y_t is the retail sales data for each week of the year, t, during 2008, α and β terms are the regression coefficients for each sine and cosine function, and ε is an error term.

The model-fitting process was repeated for each product category at each spatial resolution. This resulted in between 1 and 4 sine-cosine pairs across the different product groups. In each case, the fitted seasonal model was used to derive weekly residuals for each week of the 2009 and 2010 data; these residuals, which are normalized with respect to normal non-pandemic seasonal sales, are used in the comparative analysis (Supplementary Appendix Table A1, Figure A1 and A2).

Pearson's correlation was performed between each product category, national UK cases and media reporting. Analysis was performed for the whole pandemic period as well as the early pandemic period (06 April- 01 June 2009, media reporting only), summer pandemic wave (01 June – 30 August 2009, case and media reporting) and winter pandemic wave (31 August 2009 – 14 February 2010, case and media reporting). HPA/Q-Surveillance cases were examined at different geographic scales and evaluated by Pearson's correlation coefficients. For each product category, correlation between residual sales and cases was assessed for the period 4 May 2009 to 09 November 2009. As a rise in retail sales might be expected to occur before an outbreak is detected through healthcare based surveillance cross correlation with weekly time lags was also performed.

Spatial correlation was performed to look for evidence of clustering of residual sales and influenza cases at different time points. This was performed using the 'spatial test' function in R statistical language, included in the GeoR package [27]: this calculates a test statistic by Monte Carlo permutation testing for spatial autocorrelation based on the use of variograms. For each product group, this test statistic was calculated for sub-regional residual sales. These spatial correlations were then examined as part of the weekly time series.

All data adjustment and analysis was performed using R statistical software, version 2.15.2. Statistical significance was set at 95%.

Results

During the declared pandemic period there were two peaks of estimated cases in the summer and winter seasons seen in ~~both HPA/Q-Surveillance and~~ national flusurvey data (Figure 1). HPA/QSurveillance data at a national scale did not show a winter peak. This is most likely due to the established presence of the NPFS service which triaged influenza like illness resulting in a reduced number of primary care consultations. Media reporting was high in the early pandemic period (where there were relatively few cases in the UK) and during the summer wave but was less during the winter wave. Unadjusted national retail sales are shown in Figure 1 on a logarithmic scale.

For peer review only

There was a statistically significant positive correlation between thermometer and anti-viral product sales and national cases for the whole pandemic period (Table 2). When divided into summer and winter pandemic waves, the correlation was stronger in the summer wave than the winter wave. Children's cold and flu remedies were also positively correlated with national cases during the summer wave but not in the winter wave. Correlation between weekly residual sales and weekly media reporting was also performed (Table 2 and SA Table A1A2). Thermometer and anti-viral products were significantly positively correlated with media reporting for the whole pandemic period (Cor 0.477 (95% CI 0.171-0.699)); 0.711 (95% CI 0.495-0.844) respectively). No product group sales were significantly associated with media reporting in the early pandemic period though the strength of correlation was higher in the summer than the winter wave (Table 2 and SA Table A2).

At a regional level there was no significant correlation between estimated influenza cases and retail sales of adult 'cold and flu' remedies, cough remedies or tissues. There were weak but statistically significant correlations between sales of children's remedies and cases in six English regions and Wales (SA Table A3). Stronger positive correlations were seen between thermometer and cases and hand-gel sales and cases across all English regions and Wales (SA Table A3). No additional significant correlations were identified through cross-correlation analysis. The strongest correlation in cross-correlation testing was for no lag (0 weeks) for all comparisons.

At the sub-regional level there was a significant positive correlation between thermometer and hand-gel sales and cases in England (69.9%, 109/156; 71.8%, 112/156 respectively) (Figure 2). Several sub-regions had a statistically significant positive correlation between cases and sales of adult 'cold and flu' remedies (3.2%, 5/156) and cough remedies (3.8%, 6/156); however, a greater number of sub-regions had a significant correlation between cases and children's remedy sales (35.6%, 55/156).

We found periods of significant spatial structure throughout the pandemic period for all sale products (SA Figure A3), particularly for tissue and anti-viral products sales which appear to have more sustained periods of spatial patterning thatn the other product types.

Discussion

We analysed non-prescription retail sales data for a major GB UK supermarket in comparison with cases of pandemic influenza syndromic case estimates within Great Britain to assess the utility of purchase data to reflect case estimates from existing surveillance methods.to inform and augment existing surveillance methods. We found a poor match between symptomatic remedies and cases at the national scale for both summer and winter waves of the pandemic. However, we found a significant association between children's remedies and cases for the summer wave at the national scale, and sub-regional scales, where we found significant association in 55 out of 156 PCTs. Significant positive correlations between cases and sales of thermometers and anti-viral hand gels and hand wash were seen at all spatial scales.

One concern about the use of retail sales as a surveillance tool is that it may be more easily influenced by factors other than symptomatic cases, such as heightened media coverage, and

For peer review only

promotional activity by manufacturers, supermarkets and government, than other forms of reporting. The greatest press coverage occurred during the early pandemic period where there were relatively few cases of pandemic influenza in the GBthe UK. The lack of correlation between sales and this heightened coverage during this period suggests that 'panic buying' of symptomatic remedies or non-pharmacological groups in response to media reports did not occur. The lack of correlation between sales and media reports in the winter suggests that sales were more driven by cases than media reports as there was a similar level of cases in both the summer and winter periods.

The use of sales information for adult and child remedy products has been suggested as a useful augmentation to traditional surveillance mechanisms [8 13 14], but has not been tested within the GBthe UK. Previous studies have suggested that more localised retail sales data is more reflective of surveyed influenza patterns than national level data [11 12 28] [12]in other counties suggest that national-scale data is uninformative [12], but more localised data can reflect surveyed influenza patterns [11 25]. Our results broadly support these observations. Some products may be more useful than others in their relative ability to reflect underlying disease incidence [29]. Our results suggest that children's remedies may better reflect community infection patterns than adult products. This may be due to children being at higher risk of infection with 2009 pandemic influenza than adults [30], being more likely to be symptomatic [31 32], or may reflect adult-parent differences in self-medication practices [33]. We find no evidence that retails sales may detect cases earlier than established surveillance systems, though our analysis is limited by data resolved at a weekly scale.

Sales of anti-viral products and thermometers were highly associated with both pandemic influenza cases and media and public interest measures, especially during the first 'summer' wave of the pandemic. This finding was not replicated in tissue sales and may reflect larger unit sales per 100,000 customers making signals harder to detect. The use of anti-viral products and thermometers (for self-diagnosis) were recommended by UK government public health messaging during the early months of the pandemic and throughout the pandemic [19]. Cross sectional telephone surveys have generally reported low level of uptake of public health advice [34 35] but there is some evidence that this is a poor indicator of actual behaviour when more objective measures are used [36]. We believe our results are the first national-scale evidence that the public actively responded to these messages, at least through the purchasing of such products, and provides an alternative objective measure of public response to health advice.

There are several limitations to this study. The sales data used here are derived from the shopping purchases of a sample of shopping baskets, and only from purchases involving presentation of a loyalty card. The sales data are only sourced from one supermarket chain, and while that chain has one of the largest market shares nationally in the UK, many non prescription purchases are likely to be made in other outlets (such as dedicated pharmacies) which may better reflect community incidence of infection. The available sales data, while resolved to purchases made at an individual store level, was only available at a weekly time resolution preventing more finely resolved temporal analysis. Sales of anti-pyretic medication not branded as 'cold and flu remedies' were excluded from our analysis because of concerns regarding the interpretation of signals from these products. Remedy products may be purchased for a variety of reasons other than to directly medicate against

For peer review only

infection with influenza: they could be used for symptom alleviation for a range of other pathogen infections and conditions. We do not know if and how purchasing patterns reflect the use of the products themselves: individuals may use previously purchased products at the onset of new symptoms, only purchasing products when these expire, rather than buying new products to treat a new illness. We did not have access to surveillance data at PCT level for the full pandemic period, which would have been very valuable. The case data to which we compared the retail sales information is largely based on diagnosis of influenza-like illness cases (syndromic illness) and not virologically confirmed cases. Case data used in this analysis was not stratified by age; we were therefore unable to perform a more appropriate comparison of case data with adult and children products. Purchasing patterns made over a greater number of years and influenza seasons could have improved the seasonality estimation of purchasing behaviour.

The pandemic of 2009 was of a mild strain, which did not appear to generate a large volume of community cases which self-medicated using OTC remedies and which did not present to existing surveillance mechanisms. However, at particular spatial scales and in particular age-groups, or (we suggest) for more severe strains, retail sales may help augment existing surveillance mechanisms to provide a quantitative indication of care-seeking behaviour. However, there remain considerable uncertainties in the specific usage and self-medicating behaviour of individuals in relation to infection and purchasing of products: further investigation is required prior to the use of sales data for surveillance purposes.

Conclusions

Retail sales of over the counter symptom remedies at a national level are unlikely to be useful for the detection of cases. However, at more finely resolved spatial scales and in particular age-groups retail sales may help augment existing influenza surveillance and merit further study. Our study demonstrates that the retail sales patterns of particular product types, such as personal hygiene and self-diagnosis products, can be of value in assessing public responses to regional and national health messaging.

Contributors

ST performed the analysis and took the lead in writing; JMR conceived the study; all authors designed the analysis and commented on manuscript drafts. The authors are grateful to John Edmunds and Ellen Brooks Pollock for providing Flu Survey case estimates and to James Rubin and Susan Michie for providing aggregated media article counts. JMR would like to thank Ashleigh Jellicoe and Xu-Sheng Zhang for assistance in compiling newspaper and retail sales data.

The views expressed are those of the authors and not necessarily those of the NHS, the NIHR, the Department of Health, or Public Health England.

Funding

ST acknowledges funding from the Wellcome Trust (097465/B/11/Z). JMR acknowledges support for this work from the Economic and Social Research Council (grant ES/K004255/1) and the Medical Research Council (grant RES-355-25-0019). ~~PJW~~ PJW thanks the MRC for Centre funding, and also

For peer review only

thanks the UK National Institute for Health Research Health Protection Research Unit (NIHR HPRU) in Modelling Methodology at Imperial College London in partnership with Public Health England (PHE) for funding.thanks the Medical Research Council for centre funding.

Competing Interests: None.

Figure 1. Top Panel: Weekly estimated cases of influenza shown are from English GP surveillance system (HPA/Q-surveillance) and UK wide estimates adjusted for changes in care seeking behaviour (Flu Survey). Middle Panel: Weekly sales per 100,000 customers of six product groups from a national UK retailer. Bottom Panel: Scaled weekly estimates of UK media interest (number of relevant newspaper headlines (LexisNexis) or newspaper, radio and television articles (Meltwater)); UK public interest is represented by relative internet search volume from Google Search Trends.

Figure 2. Correlations between sales of 6 product categories and Influenza A H1N1/pdm cases during 2009. Points relate to a geographic region, size of the point and depth of colour is related to the strength of the correlation.

References

1. World Health Organisation. International Health Regulations 2nd Ed ed, 2005.
2. Ortiz JR SV, Uez OC, Oliva O, Bettels D, McCarron M, et al. Strategy to enhance influenza surveillance worldwide. Emerg Infect Dis [serial on the Internet] 2009;Available from http://wwwnc.cdc.gov/eid/article/15/8/08-1422.htm
3. Triple SP. Assessment of syndromic surveillance in Europe. The Lancet 2011;378(9806):1833-34
4. Briand S, Mounts A, Chamberland M. Challenges of global surveillance during an influenza pandemic. Public Health 2011;125(5):247-56 doi: 10.1016/j.puhe.2010.12.007[published Online First: Epub Date]|.
5. Lipsitch M, Hayden FG, Cowling BJ, Leung GM. How to maintain surveillance for novel influenza A H1N1 when there are too many cases to count. The Lancet 2009;374(9696):1209-11 doi: 10.1016/s0140-6736(09)61377-5[published Online First: Epub Date]|.
6. Severi E, Heinsbroek E, Watson C, Catchpole M. Infectious disease surveillance for the London 2012 Olympic and Paralympic Games. Euro surveillance : bulletin europeen sur les maladies transmissibles = European communicable disease bulletin 2012;17(31)
7. Dailey L, Watkins RE, Plant AJ. Timeliness of data sources used for influenza surveillance. Journal of the American Medical Informatics Association : JAMIA 2007;14(5):626-31 doi: 10.1197/jamia.M2328[published Online First: Epub Date]|.
8. Wagner MM, Tsui FC, Espino J, et al. National Retail Data Monitor for public health surveillance. MMWR. Morbidity and mortality weekly report 2004;53 Suppl:40-2
9. Welliver RC, Cherry JD, Boyer KM, et al. Sales of nonprescription cold remedies: a unique method of influenza surveillance. Pediatric research 1979;13(9):1015-7
10. Davies GR, Finch RG. Sales of over-the-counter remedies as an early warning system for winter bed crises. Clinical microbiology and infection : the official publication of the European Society of Clinical Microbiology and Infectious Diseases 2003;9(8):858-63
11. Das D, Metzger K, Heffernan R, Balter S, Weiss D, Mostashari F. Monitoring over-the-counter medication sales for early detection of disease outbreaks--New York City. MMWR. Morbidity and mortality weekly report 2005;54 Suppl:41-6

12. Ohkusa Y, Shigematsu M, Taniguchi K, Okabe N. Experimental surveillance using data on sales of over-the-counter medications--Japan, November 2003-April 2004. MMWR. Morbidity and mortality weekly report 2005;54 Suppl:47-52
13. Socan M, Erculj V, Lajovic J. Early detection of influenza like illness through medication sales. Central European journal of public health 2012;20(2):156-62
14. Goldenberg A, Shmueli G, Caruana RA, Fienberg SE. Early statistical detection of anthrax outbreaks by tracking over-the-counter medication sales. Proceedings of the National Academy of Sciences of the United States of America 2002;99(8):5237-40 doi: 10.1073/pnas.042117499[published Online First: Epub Date]|.
15. Burkom HS, Elbert Y, Feldman A, Lin J. Role of data aggregation in biosurveillance detection strategies with applications from ESSENCE. MMWR. Morbidity and mortality weekly report 2004;53 Suppl:67-73
16. Proctor ME, Blair KA, Davis JP. Surveillance data for waterborne illness detection: an assessment following a massive waterborne outbreak of Cryptosporidium infection. Epidemiology & Infection 1998;120(01):43-54
17. Kirian ML, Weintraub JM. Prediction of gastrointestinal disease with over-the-counter diarrheal remedy sales records in the San Francisco Bay Area. BMC medical informatics and decision making 2010;10:39 doi: 10.1186/1472-6947-10-39[published Online First: Epub Date]|.
18. Health Protection Agency. Epidemiological report of pandemic (H1N1) 2009 in UK. http://www.hpa.org.uk/Publications/InfectiousDiseases/Influenza/1010EpidemiologicalreportofpandemicH1N12009inUK/, 2010.
19. NHS Scotland; NHS Wales; Department of Health SSaPS. Important information about swine flu—leaflet. Last Update 04 April 2014. www.nhs.uk/news/2009/04April/Documents/Swine%20Flu%20Leaflet_Web%20Version.pdf.
20. Rutter P, Mytton O, Ellis B, Donaldson L. Access to the NHS by telephone and Internet during an influenza pandemic: an observational study. BMJ Open 2014;4(2) doi: 10.1136/bmjopen-2013-004174[published Online First: Epub Date]|.
21. Public Health England. Influenza Surveillance in the United Kingdom. Last Update. http://www.hpa.org.uk/web/HPAweb&HPAwebStandard/HPAweb_C/1195733821514.
22. Brooks-Pollock E, Tilston N, Edmunds WJ, Eames KT. Using an online survey of healthcare-seeking behaviour to estimate the magnitude and severity of the 2009 H1N1v influenza epidemic in England. BMC infectious diseases 2011;11:68
23. HarcourtT SE, Smith GE, Elliot AJ, et al. Use of a large general practice syndromic surveillance system to monitor the progress of the influenza A(H1N1) pandemic 2009 in the UK. Epidemiology & Infection 2012;140(01):100-05 doi: doi:10.1017/S095026881100046X[published Online First: Epub Date]|.
24. LexisNexis. Nexis | Home. Last Update 16 January 2014. http://www.nexis.co.uk/.
25. Meltwater. Meltwater - Online Media Monitoring - Public Relations Software - Social Marketing Software. Last Update 16 January 2014. http://www.meltwater.com/.
26. Google. Google Trends. Last Update 16 January 2014. http://www.google.com/trends/.
27. Riberio J, Diggle P. geoR: A package for geostatistical analysis. R-NEWS 2001;1(2)
28. Vergu E, Grais RF, Sarter H, et al. Medication sales and syndromic surveillance, France. Emerging infectious diseases 2006;12(3):416-21
29. Wallstrom GL, Hogan WR. Unsupervised clustering of over-the-counter healthcare products into product categories. Journal of biomedical informatics 2007;40(6):642-8 doi: 10.1016/j.jbi.2007.03.008[published Online First: Epub Date]|.
30. Miller E, Hoschler K, Hardelid P, Stanford E, Andrews N, Zambon M. Incidence of 2009 pandemic influenza A H1N1 infection in England: a cross-sectional serological study. The Lancet 2010;375(9720):1100-08

For peer review only

31. Dawood FS, Iuliano AD, Reed C, et al. Estimated global mortality associated with the first 12 months of 2009 pandemic influenza A H1N1 virus circulation: a modelling study. Lancet Infect Dis 2012;12(9):687-95 doi: 10.1016/s1473-3099(12)70121-4[published Online First: Epub Date]|.
32. Presanis AM, Pebody RG, Paterson BJ, et al. Changes in severity of 2009 pandemic A/H1N1 influenza in England: a Bayesian evidence synthesis. BMJ 2011;343:d5408 doi: 10.1136/bmj.d5408[published Online First: Epub Date]|.
33. Vernacchio L, Kelly JP, Kaufman DW, Mitchell AA. Cough and Cold Medication Use by US Children, 1999–2006: Results From the Slone Survey. Pediatrics 2008;122(2):e323-e29 doi: 10.1542/peds.2008-0498[published Online First: Epub Date]|.
34. Rubin GJ, Amlôt R, Page L, Wessely S. Public perceptions, anxiety, and behaviour change in relation to the swine flu outbreak: cross sectional telephone survey. BMJ 2009;339 doi: 10.1136/bmj.b2651[published Online First: Epub Date]|.
35. SteelFisher GK, Blendon RJ, Ward JRM, Rapoport R, Kahn EB, Kohl KS. Public response to the 2009 influenza A H1N1 pandemic: a polling study in five countries. The Lancet Infectious Diseases 2012;12(11):845-50
36. Fleischman DS, Webster GD, Judah G, de Barra M, Aunger R, Curtis VA. Sensor recorded changes in rates of hand washing with soap in response to the media reports of the H1N1 pandemic in Britain. BMJ Open 2011;1(2) doi: 10.1136/bmjopen-2011-000127[published Online First: Epub Date]|.

For peer review only

Table 1: Data Sources of Influenza Case Estimates, Media Reporting and Public Interest

Data	Description	Source	Dates	Ref
Flu Survey	~~GB~~ UK National Case Estimates	Adjusted healthcare-based surveillance system.	01 June 2009 - 08 Feb 2010	[22]
HPA/Q Surveillance	Regional Case Estimates	General Practitioner symptomatic surveillance	04 May 2009 – 15 Nov 2009	[23]
LexisNexis	UK Media Coverage	UK newspaper headlines with reference to A/H1N1pdm and related terms	25 April 2009 – 27 Dec 2009	[24]
Meltwater	UK Media Coverage	UK newspaper headlines, radio and television news items with reference to A/H1N1pdm and related terms	06 April 2009 – 19 April 2010	[25]
Google Trends	UK Internet Searches	Internet searches from UK IP addresses with reference to A/H1N1pdm and related terms	06 April 2009 - 28 Dec 2009	[26]

For peer review only

Table 2: Correlation between Retail Sales, National Cases and Media Interest * <0.05; ** <0.01 ***<0.001

	Whole Pandemic Period (19 April 2009–14 Feb 2010)		*Early Pandemic (19 April -31 May 2009)*		*Summer Wave (01 June – 30 Aug20 09)*		*Winter Wave (31 Aug 2009 – 14 Feb 2010)*	
	**Cor**	**95% CI**	**Cor**	**95% CI**	**Cor**	**95% CI**	**Cor**	**95% CI**
	**FluSurvey Case Estimates**							
**ADULT COLD & FLU REMEDIES**	0.116	-0.216 0.424	-	-	0.193	-0.401 0.672	0.149	-0.270 0.521
**CHILDRENS' COLD & FLU REMEDIES**	-0.023	-0.344 0.303	-	-	0.778**	0.396 0.930	0.010	-0.395 0.412
**COUGH REMEDIES**	0.374*	0.056 0.622	-	-	0.245	-0.353 0.702	0.396	-0.009 0.689
**THERMOMETERS**	0.445**	0.142 0.672	-	-	0.935***	0.792 0.981	0.796***	0.579 0.908
**ANTI-VIRAL PRODUCTS**	0.072	-0.258 0.387	-	-	0.671*	0.190 0.892	0.014	-0.392 0.415
**TISSUES**	0.051	-0.278 0.369	-	-	0.128	-0.455 0.634	-0.057	-0.450 0.354
	**Meltwater Reports**							
**ADULT COLD & FLU REMEDIES**	-0.256	-0.488 0.010	-0.379	-0.855 0.444	0.151	-0.436 0.648	-0.399	-0.691 0.005
**CHILDRENS' COLD & FLU REMEDIES**	0.171	-0.099 0.417	0.447	-0.376 0.876	0.576*	0.037 0.856	-0.427*	-0.708 -0.029
**COUGH REMEDIES**	-0.225	-0.462 0.043	-0.447	-0.876 0.376	0.249	-0.350 0.703	-0.129	-0.506 0.290
**THERMOMETERS**	0.364**	0.110 0.574	0.374	-0.449 0.854	0.772**	0.384 0.928	0.378	-0.030 0.678
**ANTI-VIRAL PRODUCTS**	0.458***	0.219 0.645	0.537	-0.270 0.901	0.516	-0.049 0.831	-0.119	-0.498 0.299
**TISSUES**	-0.288	-0.514 -0.025	0.386	-0.437 0.858	0.241	-0.358 0.699	-0.451	-0.723 -0.059
	**Google Searches**							
**ADULT COLD & FLU REMEDIES**	0.051	-0.269 0.360	-0.241	-0.808 0.559	0.258	-0.341 0.708	-0.214	-0.619 0.281
**CHILDRENS' COLD & FLU REMEDIES**	0.369*	0.060 0.613	0.452	-0.371 0.877	0.716**	0.273 0.909	-0.303	-0.674 0.191
**COUGH REMEDIES**	-0.050	-0.360 0.270	-0.318	-0.836 0.498	0.295	-0.306 0.728	-0.083	-0.529 0.399
**THERMOMETERS**	0.661***	0.437 0.808	0.212	-0.579 0.797	0.891***	0.669 0.967	0.570*	0.140 0.819
**ANTI-VIRAL PRODUCTS**	0.562***	0.299 0.745	0.346	-0.474 0.845	0.610*	0.089 0.869	0.038	-0.437 0.496
**TISSUES**	-0.063	-0.371 0.257	0.196	-0.590 0.791	0.296	-0.305 0.728	-0.034	-0.493 0.440

For peer review only

~~**Figure 1**. Top Panel: Weekly estimated cases of influenza shown are from English GP surveillance system (HPA/Q surveillance) and UK wide estimates adjusted for changes in care seeking behavior (Flu Survey). Middle Panel: Weekly sales per 100,000 customers of six product groups from a national UK retailer. Bottom Panel: Scaled weekly estimates of UK media interest (number of relevant newspaper headlines (LexisNexis) or newspaper, radio and television articles (Meltwater)); UK public interest is represented by relative internet search volume from Google Search Trends.~~

~~**Figure 2**. Correlations between sales of 6 product categories and Influenza A H1N1/pdm cases during 2009. Points relate to a geographic region, size of the point and depth of colour is related to the strength of the correlation.~~

For peer review only

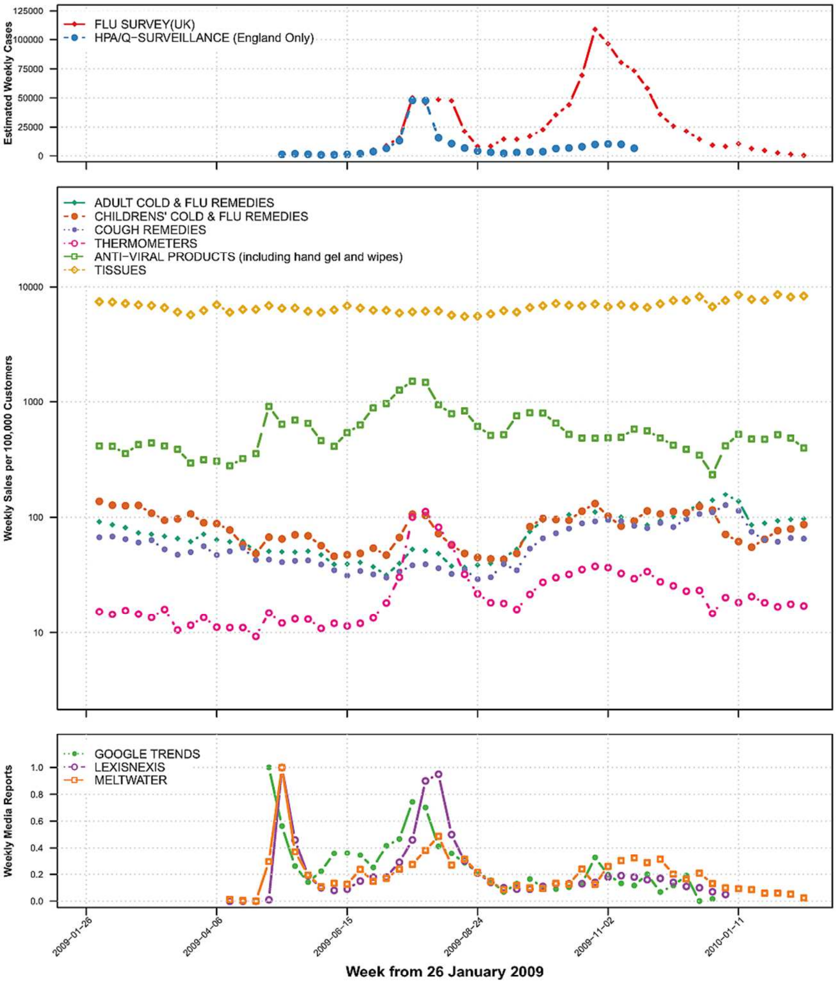

90x127mm (300 x 300 DPI)

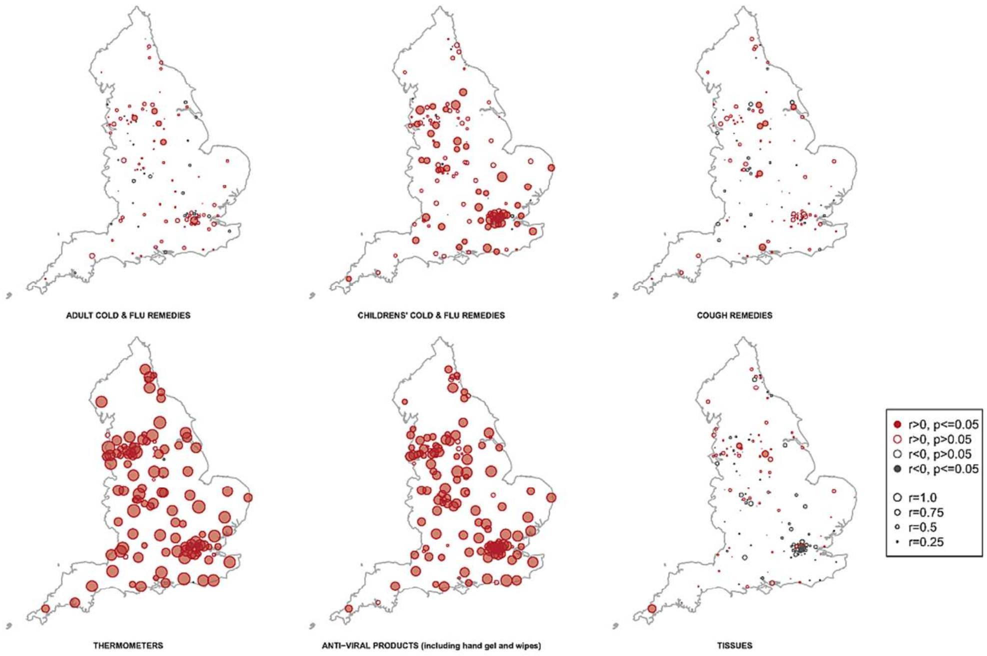

90x63mm (300 x 300 DPI)

view only

Table A1: Number of Sine/Cosine Pairs in Model Fit by Product Group and Region

	1 Sine/Cosine Pair	*2 Sine/Cosine Pair*	*3 Sine/Cosine Pair*	*4 Sine/Cosine Pair*	*Unable to Fit*
**National**					
**ADULT COLD & FLU REMEDIES**	0	0	1	0	0
**CHILDRENS' COLD & FLU REMEDIES**	0	0	1	0	0
**COUGH REMEDIES**	0	0	1	0	0
**THERMOMETERS**	0	0	1	0	0
**ANTI-VIRAL PRODUCTS**	1	0	0	0	0
**TISSUES**	0	1	0	0	0
**Regional**					
**ADULT COLD & FLU REMEDIES**	0	1	11	0	1
**CHILDRENS' COLD & FLU REMEDIES**	2	3	7	1	0
**COUGH REMEDIES**	0	0	12	0	1
**THERMOMETERS**	0	8	5	0	0
**ANTI-VIRAL PRODUCTS**	13	0	0	0	0
**TISSUES**	0	13	0	0	0
**Subregional**					
**ADULT COLD & FLU REMEDIES**	39	39	37	6	30
**CHILDRENS' COLD & FLU REMEDIES**	88	37	24	1	1
**COUGH REMEDIES**	51	40	4	1	30
**THERMOMETERS**	108	14	4	1	24
**ANTI-VIRAL PRODUCTS**	143	6	2	0	0
**TISSUES**	41	105	4	1	0

For peer review only

Table A2: Correlation between Retail Sales, and Media Interest * <0.05; ** <0.01 ***<0.001

	Whole Pandemic Period (19 April 2009–14 Feb 2010)		*Early Pandemic (19 April -31 May 2009)*		*Summer Wave (01 June – 30 Aug2009)*		*Winter Wave (31 Aug 2009 – 14 Feb 2010)*	
	**Cor**	**95% CI**	**Cor**	**95% CI**	**Cor**	**95% CI**	**Cor**	**95% CI**
	**LexisNexis**							
**ADULT COLD & FLU REMEDIES**	0.169	-0.174 0.476	-0.872	-0.992 0.043	0.737**	0.313 0.916	0.171	-0.337 0.602
**CHILDRENS' COLD & FLU REMEDIES**	0.452**	0.140 0.682	-0.890*	-0.993 - 0.038	0.870***	0.612 0.960	0.101	-0.399 0.555
**COUGH REMEDIES**	-0.015	-0.347 0.319	-0.856	-0.990 0.107	0.760*	0.359 0.924	0.242	-0.270 0.647
**THERMOMETERS**	0.477**	0.171 0.699	0.536	-0.657 0.963	0.799**	0.444 0.937	0.559*	0.108 0.820
**ANTI-VIRAL PRODUCTS**	0.711***	0.495 0.844	0.786	-0.314 0.985	0.853***	0.569 0.955	0.212	-0.299 0.629
**TISSUES**	-0.149	-0.460 0.194	0.861	-0.089 0.991	-0.088	-0.610 0.486	0.141	-0.364 0.583

For peer review only

Table A3: Correlation between Retail Sales and Regional Cases * <0.05; ** <0.01 ***<0.001

	ADULT COLD & FLU REMEDIES		CHILDRENS' COLD & FLU REMEDIES		COUGH REMEDIES		THERMOMETERS		ANTI-VIRAL PRODUCTS (including hand gel and wipes)		TISSUES	
	Cor	95% CI	Cor	95% CI	Cor	95% CI	Cor	95% CI	Cor	95% CI	Cor	95% CI
South Central	0.211	-0.176 0.542	0.376*	0.003 0.657	0.128	-0.257 0.478	0.905***	0.803 0.955	0.673***	0.401 0.836	-0.012	-0.384 0.362
East Of England	0.049	-0.330 0.414	0.354	-0.022 0.643	0.034	-0.344 0.402	0.923***	0.838 0.964	0.778***	0.571 0.892	-0.082	-0.442 0.300
London	0.155	-0.232 0.499	0.553**	0.226 0.767	0.331	-0.048 0.627	0.860***	0.717 0.934	0.792***	0.595 0.900	-0.206	-0.538 0.181
South East Coast	0.163	-0.223 0.506	0.590**	0.278 0.789	0.077	-0.304 0.438	0.925***	0.842 0.965	0.768***	0.554 0.887	-0.052	-0.417 0.328
South West	0.105	-0.279 0.460	0.389*	0.019 0.666	0.003	-0.371 0.375	0.924***	0.840 0.964	0.658***	0.378 0.828	0.024	-0.352 0.394
North West	0.099	-0.284 0.456	0.189	-0.198 0.525	0.055	-0.325 0.419	0.934***	0.862 0.969	0.710***	0.459 0.856	0.082	-0.300 0.442
East Midlands	0.145	-0.242 0.491	0.323	-0.056 0.622	0.018	-0.357 0.388	0.926***	0.845 0.966	0.840***	0.680 0.924	0.046	-0.333 0.412
West Midlands	-0.089	-0.447 0.294	0.281	-0.103 0.592	0.030	-0.347 0.399	0.863***	0.723 0.935	0.795***	0.600 0.901	-0.025	-0.394 0.351
Yorkshire And The Humber	0.241	-0.145 0.563	0.437*	0.077 0.697	0.194	-0.193 0.529	0.926***	0.845 0.966	0.721***	0.477 0.862	0.147	-0.240 0.493
Wales	0.151	-0.235 0.496	0.418*	0.053 0.684	0.125	-0.260 0.476	0.944***	0.882 0.974	0.509**	0.168 0.741	0.216	-0.171 0.545
North East	0.268	-0.117 0.583	0.510**	0.169 0.742	0.227	-0.160 0.553	0.945***	0.884 0.975	0.728***	0.487 0.866	0.032	-0.345 0.400

For peer review only

Figure A1. For each geographic region and each product category a trend of weekly sales in 2008-2010 is available (grey line). A trend of seasonal sales is calculated from 2008 sales (trend line to left of the vertical black dotted line). The optimal number of sine/cosine pairs is selected using a maximised log likelihood method. From that a fitted line of the expected seasonal sales for that product group at that spatial resolution is generated (dashed red line). This seasonal trend acts as the 'expected baseline sales' for each corresponding week of 2009 and 2010. The residual sales are used within the analysis. National UK data is shown in this figure.

For peer review only

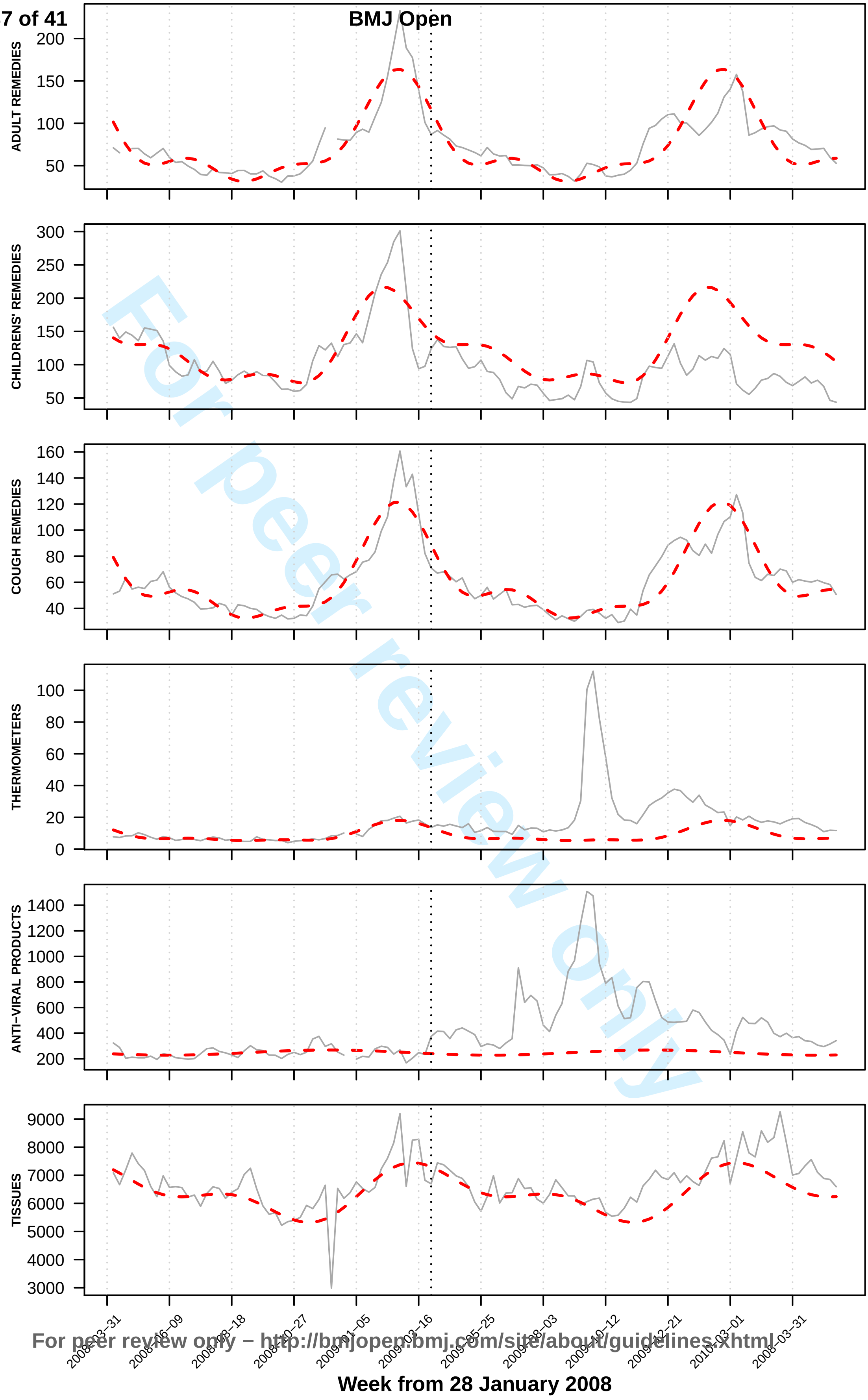

For peer review only

Figure A2. The optimal number of sine/cosine pairs for fitted line is calculated using a log likelihood method. This figure demonstrates the differences in optimal fitted lines (dashed red line) for different sub-regions based on 2008 sales of childrens' remedies (grey line to left of vertical black dotted line).

For peer review only

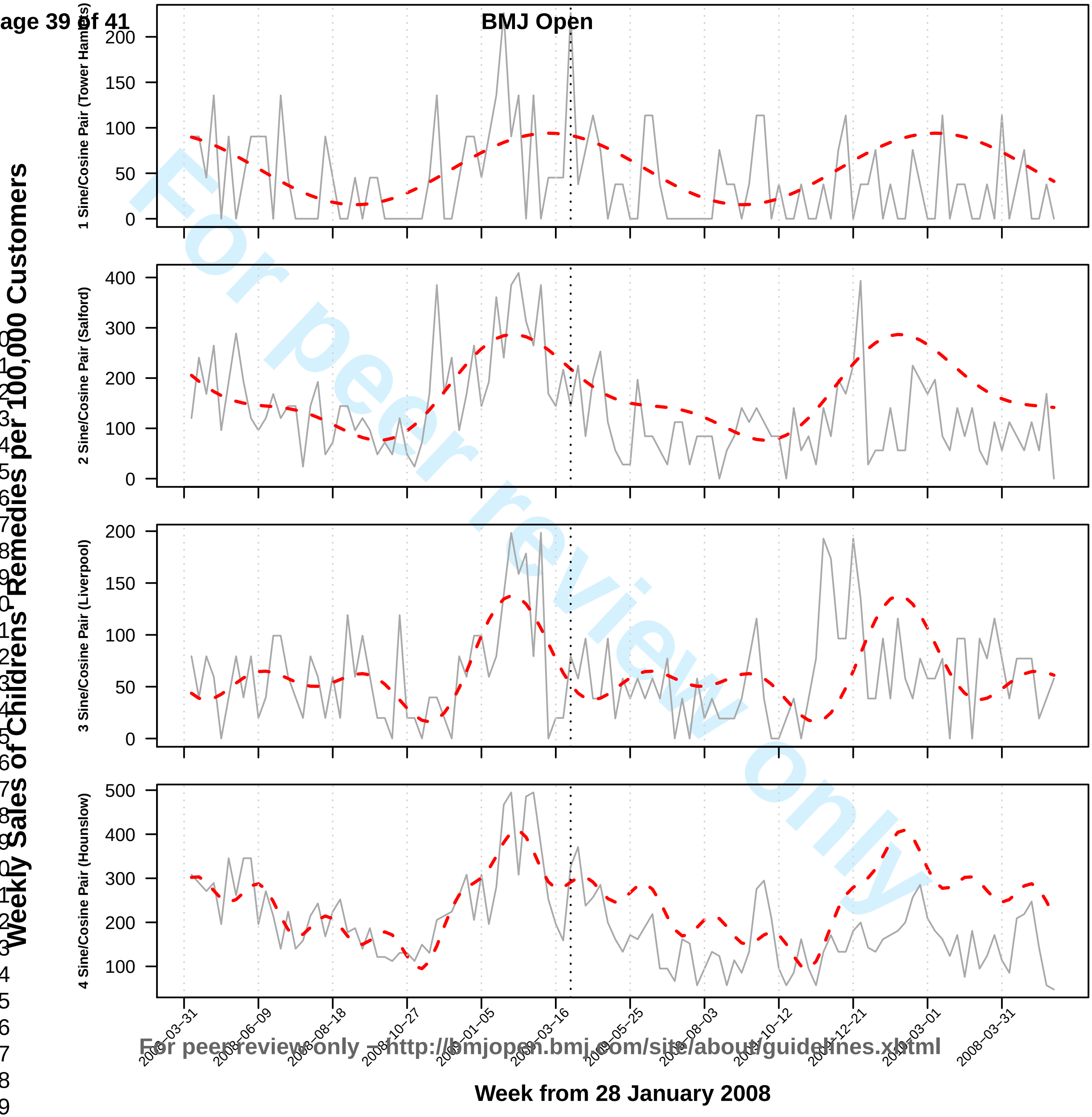

For peer review only

Figure A3. Test for spatial structure in sales of six product categories (whole time period) and cases of Influenza A H1N1 pdm (time period of available sub-regional case data is highlighted in red) at a sub-regional (PCT) level. This test was performed across 156 sub-regions for each week of the time period. A grey square indicates evidence of statistically significant spatial heterogeneity for the sales of that product group during that week. Darker grey indicates greater statistical significance. The coloured background regions indicate general specific periods of influenza activity (pink: early pandemic period, blue: summer pandemic wave; green: winter pandemic peak and seasonal peak in 2008.

For peer review only

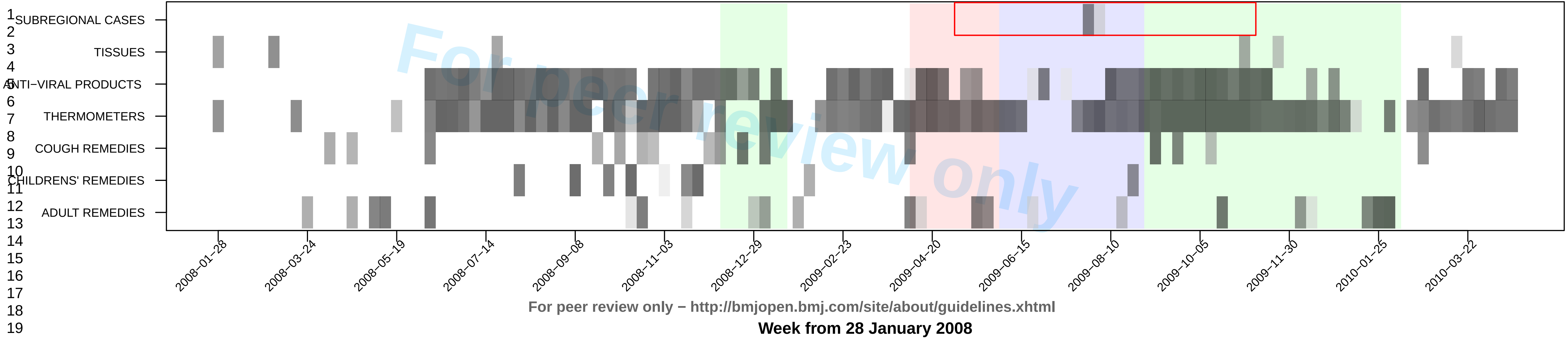

For peer review only − http://bmjopen.bmj.com/site/about/guidelines.xhtml
